# The worldwide seroprevalence of DENV, CHIKV and ZIKV infection: A systematic review and meta-analysis

**DOI:** 10.1371/journal.pntd.0009337

**Published:** 2021-04-28

**Authors:** Zhihui Li, Jin Wang, Xiaomin Cheng, Huan Hu, Cheng Guo, Jingyi Huang, Zeliang Chen, Jiahai Lu

**Affiliations:** 1 School of Public Health, Sun Yat-Sen University, Guangzhou, Guangdong Province, China; 2 Center for Infection and Immunity, Mailman School of Public Health, Columbia University, New York City, New York, United States of America; 3 Songgang People’s Hospital of Bao’an District, Shenzhen, Guangdong Province, China; Faculty of Science, Mahidol University, THAILAND

## Abstract

**Background:**

As the three major arthropod-borne viruses, dengue virus (DENV), chikungunya virus (CHIKV), and zika virus (ZIKV) are posing a growing threat to global public health and socioeconomic development. Our study aimed to systematically review the global seroprevalences of these arboviruses from existing publications.

**Methods:**

Articles published between Jan 01, 2000 and Dec 31, 2019 in the databases of Embase, Pubmed and Web of Science were searched and collected. Countries or areas with known local presence of *Aedes* vector mosquitoes were included. Random effects model was utilized to estimate the pooled seroprevalences and the proportion of inapparent infection.

**Results:**

Out of 1375, a total of 133 articles involving 176,001 subjects were included for our analysis. The pooled seroprevalences of DENV, CHIKV and ZIKV were 38%, 25% and 18%, respectively; and their corresponding proportions of inapparent infections were 80%, 40% and 50%. The South-East Asia Region had the highest seroprevalences of DENV and CHIKV, while the Region of the Americas had the highest seroprevalence of ZIKV. The seroprevalences of DENV and CHIKV were similar when comparing developed and developing countries, urban and rural areas, or among different populations. In addition, we observed a decreased global seroprevalences in the new decade (2010–2019) comparing to the decade before (2000–2009) for CHIKV. For ZIKV, the positive rates tested with the nucleic acid detection method were lower than those tested with the antibody detection method. Lastly, numerous cases of dual seropositivity for CHIKV and DENV were reported.

**Conclusions:**

Our results revealed a varied prevalence of arbovirus infections in different geographical regions and countries, and the inapparent infection accounted an unneglected portion of infections that requires more attention. This study will shed lights on our understanding of the true burden of arbovirus infections and promote appropriate vaccination in the future.

## Author summary

DENV, CHIKV, and ZIKV infections are the three major neglected tropical diseases continually posing threats to the public health and the socioeconomic development in recent decades. Given a high fraction of inapparent infections of these viruses, it is necessary to obtain informative knowledge of the seroepidemiology of DENV, CHIKV, and ZIKV worldwide, so as to estimate the true burden of these diseases and evaluate effects on vaccine efficacy and uptake. Given the fact that the reported infection rates may vary greatly due to the measurement, criteria of inclusion, and etc, a systematic review with meta-analysis is essential to collect and summarize the seroepidemiologic evidence of these arboviruses. In this study, the geographical distributions of DENV, CHIKV, and ZIKV and the proportions of their inapparent infections were analyzed, which revealed an overall high disease burden to the world, especially in specific regions. The epidemiological features of these arboviruses were also characterized via comparisons made in different countries/areas, populations, and time periods. In addition, evidence on the co-infection of DENV and CHIKV was assessed.

## Introduction

Arthropod-borne viruses (arboviruses), including dengue virus (DENV), chikungunya virus (CHIKV) and zika virus (ZIKV), continually present a threat to the global public health as well as the socioeconomic development in recent decades. These viruses are transmitted to human mostly by mosquito *Aedes aegypti* and *Aedes albopictus* all over the world [1,2]. As the most common mosquito-borne disease, dengue has become endemic in 129 countries and has caused almost 390 million infections per year [3–6]. According to the Global Burden of Disease study, dengue is spreading more rapidly than any other infectious diseases, with a 4-fold increase within 13 years (2000–2013) [5]. CHIKV has become a global threat owing to the severe debilitating nature of the disease and its unprecedented scale since a new mutant was discovered in Caribbean countries and territories in 2013 [7]. To date, human cases of chikungunya have been found in more than 100 countries [8]. ZIKV is another mosquito-transmitted flavivirus that has aroused global attention because of its potential to trigger explosive epidemics, risk of developing congenital abnormalities during pregnancy, and neurologic morbidities, such as Guillain-Barre syndrome [9,10]. As of 2017, more than 220,000 confirmed and 580,000 suspected ZIKV cases were reported in 52 countries or territories in the Americas [9]. Unfortunately, no specific treatments or effective vaccines is available to combat these aforementioned arboviruses yet, and the progress towards medicine and vaccine development is slow [3,8,11,12].

These three mosquito-borne diseases are mainly endemic in tropical and subtropical regions. However, with the population growth and urbanization, global travel and trade, vector adaption and climate change in recent decades, these infections have spread to southern China in East Asia, countries in the Pacific Ocean and the Americas, even European countries [13]. Travellers have played an important role in worldwide transmission of mosquito-borne viruses [14], as international travel can introduce both the *Aedes* and the arboviruses to new regions, leading to autochthonous outbreaks [15–17]. A study demonstrates that dengue is one of the causes of fever in travellers visiting dengue-endemic countries with about 66.2% confirmed [18].

Understanding of geographical distribution and prevalence rates of arboviruses is indispensable for estimating the true burden of these diseases as well as hypothesizing effects on vaccine efficacy and uptake. Such data are not only essential for arousing further discussion about the global impact of these mosquito-borne diseases, but also help guide scholars and policymakers to assess and identify cost-effective control strategies for disease prevention and control.

The disparity between the number of reported cases and estimates of actual cases makes it tough to capture the true burden of these mosquito-borne diseases. Approximately 80% of individuals infected by DENV and ZIKV remain asymptomatic throughout the infection, presenting no symptoms or clinical signs of infection [4,19–22], which lead to under-estimation and under-reporting by official passive surveillance and reporting systems. Seroprevalence studies, as a supplement for traditional symptom-based and laboratory-based surveillance, are essential for assessing the true disease burden of mosquito-borne diseases. In the present research, we performed a systematic review and meta-analysis to determine the seroprevalence of DENV, CHIKV, and ZIKV derived antibodies and to reveal the epidemiological characteristics of these mosquito-borne diseases via comparisons made in different countries/areas, population and time periods.

## Methods

### Literature search

This study was conducted and reported in line with the Preferred Reporting Items for Systematic Reviews and Meta-analyses (PRISMA) guidelines [23]. We searched articles published through Jan 01, 2000 to Dec 31, 2019 from the Embase, Pubmed and Web of Science. The keywords were determined through searching in the Medical Subject Headings (MeSH) database. The search terms were: (“chikungunya” OR “CHIKV” OR “CHIK” OR “CHIKF” OR “dengue” OR “DENV” OR “DENF” OR “Breakbone” OR “ZIKV” OR “zikv” OR “Arboviruses” OR “arbovirus” OR “arboviral” OR “mosquito-borne” OR “Arthropod-Borne”) And (“seroprevalence” OR “serosurvey” OR “serologic” OR “serological” OR “seroepidemiologic” OR “seroepidemiology” OR “prevalence”), restricted in the article title. The searched studies were limited to humans and English language publications after the year 2000. The additional relevant papers were also manually searched from the reference lists of the included publications. All publications were imported and cataloged in Endnote X9.

### Inclusion and exclusion criteria

Studies on the seroprevalence of DENV, CHIKV or ZIKV were included. Only articles published in English, focused on human, with study samples ≥ 100, and reporting at least one outcome of interest were evaluated. The exclusion criteria were 1) duplicate articles or those evaluating the same samples; 2) reviews, abstracts from conferences, dispatches, case reports, short reports, short communications and letters to the editors; 3) studies without epidemiological methods; 4) studies on febrile patients, suspected or confirmed cases, which might introduce biases in the estimation of seroprevalence; 5) abstracts or full-text not available. Additionally, countries without *Aedes* mosquitoes were excluded, such as Russia, Australia, and countries in North Europe, North America, and etc.

### Literature selection and data extraction

The studies searched were reviewed by reading titles and abstracts by two independent investigators (ZL and XC). Any discrepancies were discussed, with adjudication by a third reviewer if necessary (HH). The full article of each potentially eligible study was retrieved. The data extraction was performed independently and in duplicate by two reviewers (ZL and XC) using a standardized data extraction Excel form. Disagreements between the reviewers were resolved by discussion with the third reviewer (HH).

The following data from each study were recorded: first author, publication year, study country and/or area, recruitment method, WHO region, study period and population, sampling method, laboratory assays, age of participants, male proportion, seroprevalence, and proportions of inapparent infections. For cohort studies or those presented in more than one report, data with the largest sample size or more complete details were recorded. For the article reporting seroprevalences of different populations or countries (or areas), it was split accordingly for further analysis.

### Study quality assessment

The quality of the studies was evaluated using the Agency for Healthcare Research and Quality (AHRQ) with an 11-item checklist, as this tool has been recommended for assessment of cross-sectional/prevalence studies [24]. Each item was scored “0” for “no” or “unclear” and scored “1” for “yes”. A score of 0–3 was considered as low quality, 4–7 moderate quality, and 8–11 high quality. The assessment was performed independently and in duplicate by two reviewers (ZL and XC). Disagreements between the reviewers were resolved by discussion with the third reviewer (HH).

### Statistical analysis

In this study, the seroprevalences of DENV, CHIKV, and ZIKV were assessed globally and by WHO region. Also, for subgroup analysis, seroprevalence was evaluated separately for urban versus rural areas, children/adolescents (aged <18 years) versus adults (aged ≥18 years), general population versus special population (blood donors and pregnant women), the period of 2000–2009 versus the period of 2010–2019, as well as developed countries versus developing countries according to the United Nations Statistics Division [25]. The proportions of inapparent infections for DENV, CHIKV, and ZIKV were also analyzed.

The extracted data from eligible studies were analyzed by using STATA statistical package (StataCorp, College Station, TX, USA). Heterogeneity was checked using the *I*^*2*^ index. Random effects model and fixed effects model were chosen when *I*^*2*^ was ≥ 50% and <50%, respectively. The coverage of 95% confidence intervals (CI) was assessed to determine the significant difference among groups [26]. The map presenting the worldwide seroprevalence was made by ESRI ArcMap 10.4.1 Software.

## Results

### Study selection

**Fig 1:**
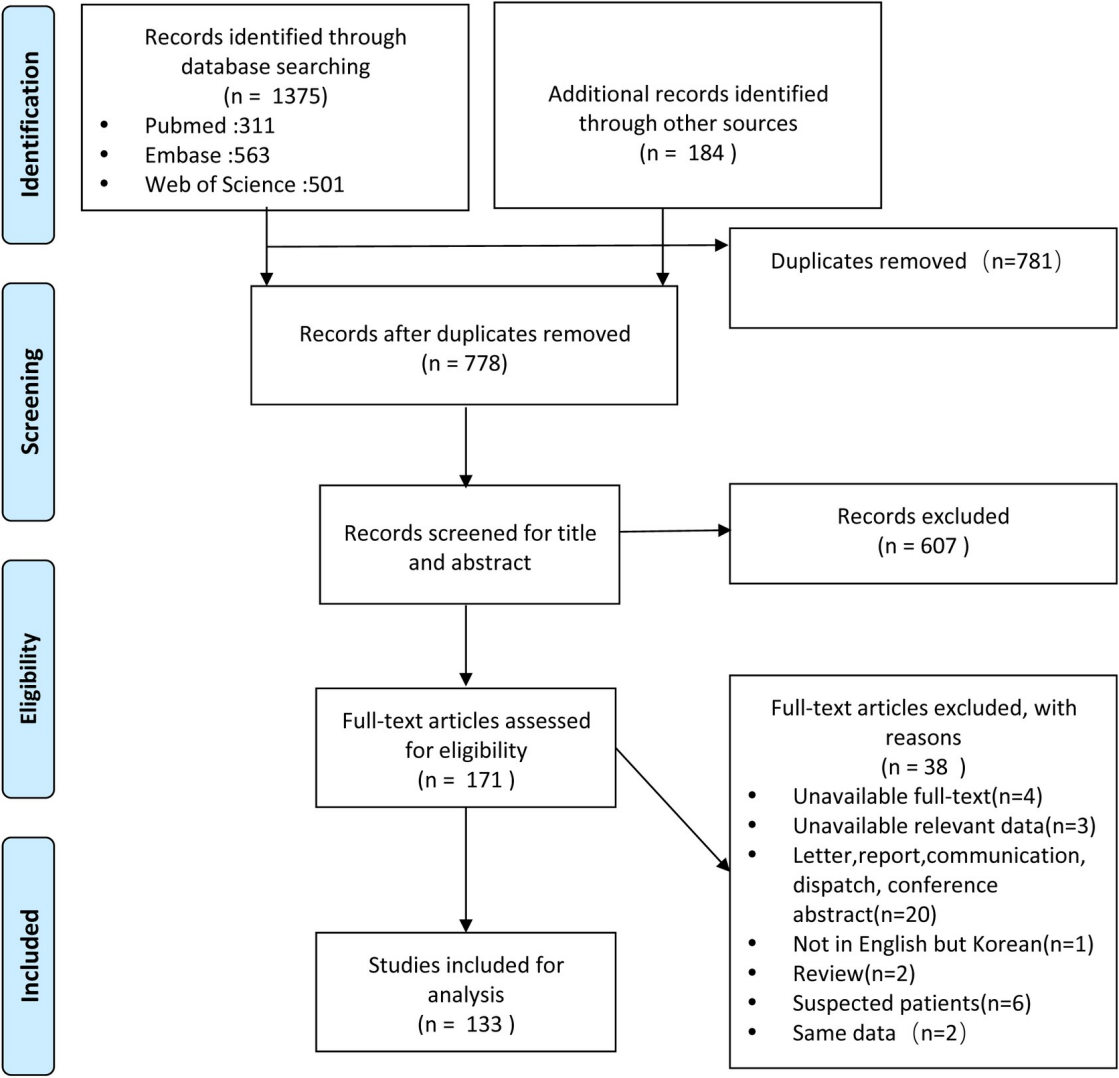
Flow diagram of publication selection process.

A total of 1375 citations were retrieved from PubMed (n = 311), Embase (n = 563), and Web of Science (n = 501), and additional records identified through other sources (n = 184), of which 781 articles were found to be duplicates. Of the 778 articles screened, there were 607 irrelevant articles excluded after reading the titles and abstracts. After the review of full-text articles, 38 articles met the exclusion criteria. And finally, a total of 133 articles were included. Fig 1 shows a PRISMA flow-chart of the study selection process (Fig 1).

### Quality of the studies

This meta-analysis included 50 studies (37.6%) with high quality of evidence and 78 studies (58.6%) with moderate quality. Only 3.8% (5/133) of studies included were considered low quality (Tables A, B, and C in S1 Text and A in S5 Text).

### General study characteristics

Of total 133 articles involving 176,001 subjects in 143 reports (DENV 87, CHIKV 30, ZIKV 11, DENV & CHIKV 12, DENV & ZIKV 1, DENV & CHIKV & ZIKV 2), geographic study locations comprised: 46 reports from Region of the Americas, 27 from Western Pacific Region, 25 from African Region, 19 from South-East Asia Region, 6 from European Region, 17 from Eastern Mediterranean Region, 3 from travellers to tropical or subtropical areas. The geographic characteristics of included studies were summarized in Tables A, B, and C in S1 Text.

There were a variety of assays used for serological diagnosis across studies. The most commonly used assay was enzyme-linked immunosorbent assay (ELISA; 89.5%, 119/133), followed by neutralization assay (NA; 6.0%, 8/133) and immunofluorescence assay (IFA; 3.8%, 5/133). Other assays included hemagglutination inhibition (HI), microsphere immunoassays (MIA), multiplex bead assay (MBA) and blockade-of-binding (BOB) assay, or combination of assays such as ELISA&NA, IFA&NA, ELISA&IFA.

### Seroprevalence of DENV infection and subgroup analysis

**Fig 2:**
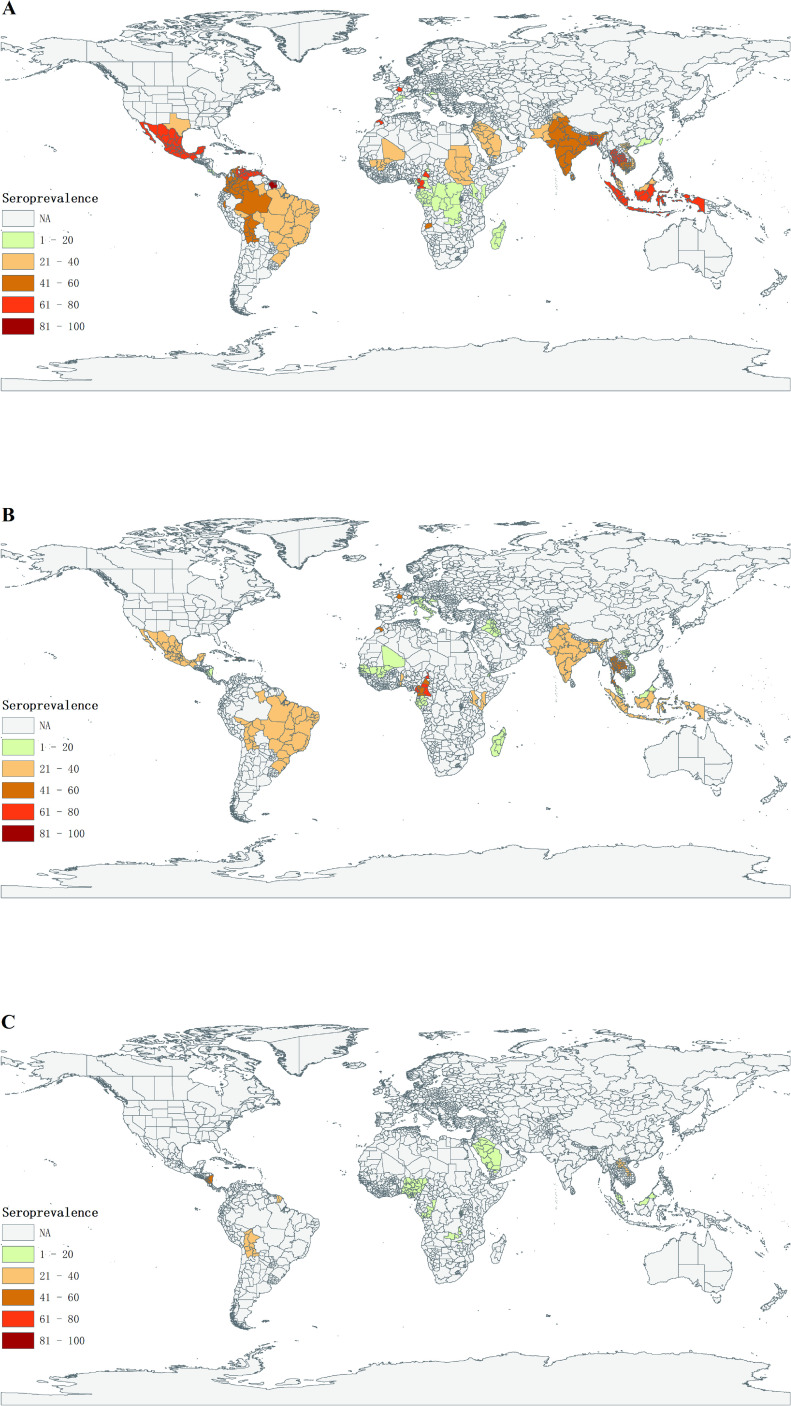
Graphical presentation of seroprevalence across the world for DENV(A), CHIKV(B) and ZIKV (C) infection. **The map was drawn by ArcGIS program (version 10.4.1; ESRI, Redlands, California, USA) using the base layer (**https://pro.arcgis.com/zh-cn/pro-app/latest/help/mapping/properties/plate-carree.htm).

The seroprevalence of DENV infection was reported in 102 studies consisted of 124,013 individuals from 44 countries and areas. And the pooled seroprevalence was 38% (95% CI: 32–43) (Table 1). The prevalence rate of DENV infection across the world is shown in Fig 2A. Among regions tested, the South-East Asia Region had the highest seroprevalence among all WHO regions (56%, 95% CI: 39–73); In contrast, European Region had the lowest seroprevalence of DENV (4%, 95% CI: 0–7).

**Table 1:** Seroprevalence of DENV, CHIKV and ZIKV infection.

WHO Region	Nations or Areas	DENV				WHO Region	Nations or Areas	CHIKV				WHO Region	Nations or Areas	ZIKV			
		Reports	Cases	Samples	Seroprevalence			Reports	Cases	Samples	Seroprevalence			Reports	Cases	Samples	Seroprevalence
		(n)	(n)	(n)	(%, 95% CI)			(n)	(n)	(n)	(%, 95% CI)			(n)	(n)	(n)	(%, 95% CI)
**African**		**10**	**1308**	**11955**	**13(9–17)**	**African**		**15**	**3463**	**13359**	**33(24–41)**	**African**		**5**	**317**	**7161**	**4(2–7)**
	**Cameroon**	1	32	256	13(8–17)		**Benin**	1	133	352	38(33–43)		**Nigeria**	1	48	468	10(8–13)
	**Congo**	1	37	978	4(3–5)		**Cameroon**	2	214	361	68(25–100)		**Madagascar**	1	6	1216	0(0–1)
	**Gabon**	1	20	162	12(7–17)		**Comoros**	1	209	331	63(58–68)		**Rwanda**	1	12	874	1(1–2)
	**Kenya**	3	515	3732	14(13–15)		**Gabon**	1	1	162	1(0–2)		**Congo**	1	34	978	3(2–5)
	**Madagascar**	1	97	1244	8(6–9)		**Kenya**	4	1094	4186	34(10–58)		**Zambia**	1	217	3625	6(5–7)
	**Mali**	1	196	805	24(21–27)		**Madagascar**	1	154	1244	12(11–14)	**Eastern Mediterranean**		**1**	**52**	**410**	**13(9–16)**
	**Zambia**	1	149	3624	4(3–5)		**Mali**	1	50	805	6(5–8)		**Saudi Arabia**	1	52	410	13(9–16)
	**Mayotte**	1	262	1154	23(20–25)		**Senegal**	1	39	1434	3(2–4)	**the Americas**		**5**	**2902**	**8962**	**34(24–45)**
Eastern* Mediterranean		**15**	**5095**	**17912**	**27(21–34)**		**Mayotte**	1	440	1154	38(35–41)		America**	1	114	367	31(26–36)
	**Sudan**	4	587	2583	22(5–40)		**La Réunion Island**	2	1129	3330	29(8–50)		**Bolivia**	1	213	814	26(23–29)
	**Djibouti**	1	199	911	22(19–25)	**Eastern Mediterranean**		**3**	**108**	**3305**	**2(0–5)**		**Nicaragua**	2	1947	4814	46(26–66)
	**Jordan**	1	219	892	25(22–27)		**Djibouti**	1	24	914	3(2–4)		**French Guiana**	1	628	2697	23(22–25)
	**Kuwait**	1	70	499	14(11–17)		**Iraq**	1	2	399	1(0–1)	**Western Pacific**		**3**	**336**	**2131**	**18(4–31)**
	**Pakistan**	2	169	640	26(23–30)		**Qatar**	1	82	1992	4(3–5)		**Laos**	2	254	1046	23(12–33)
	**Qatar**	1	473	1992	24(22–26)	**European**		**3**	**44**	**1833**	**3(1–5)**		**Malaysia**	1	82	1085	8(6–9)
	**Saudi Arabia**	5	3378	10395	37(27–46)		**Croatia**	1	9	1008	1(0–1)	**Overall**		**14**	**3607**	**18394**	**18(12–25)**
**European**		**3**	**56**	**2100**	**4(0–7)**		**Italy**	1	33	325	10(7–13)	**Inapparent** proportion		**3**	**541**	**790**	**50(18–82)**
	**Croatia**	1	7	1180	1(0–1)		**Turkey**	1	2	500	0(0–1)						
	**France**	1	17	562	3(2–4)	**South-East Asia**		**6**	**1291**	**4924**	**42(17–67)**						
	**Portugal(Madeira)**	1	32	358	9(6–12)		**India**	3	763	3571	38(0–77)						
**South-East Asia**		**17**	**15329**	**29882**	**56(39–73)**		**Indonesia**	1	77	199	39(32–45)						
	**Bangladesh**	1	900	1125	80(78–82)		**Thailand**	2	451	1154	49(6–92)						
	**India**	9	9528	21510	46(22–69)	**the Americas**		**14**	**6341**	**23394**	**24(15–34)**						
	**Indonesia**	1	2216	3194	69(68–71)		America**	3	418	1907	19(0–39)						
	**Sri Lanka**	3	1671	2799	51(33–70)		**Bolivia**	1	102	449	23(19–27)						
	**Thailand**	3	1014	1254	79(68–90)		**Brazil**	3	449	1393	24(0–61)						
**the Americas**		**33**	**15917**	**26944**	**53(39–67)**		**Haiti**	1	2570	4438	58(56–59)						
	America**	5	296	840	36(0–77)		**Mexico**	1	114	387	29(25–34)						
	**Bolivia**	1	223	449	50(45–54)		**Nicaragua**	2	316	4210	10(3–16)						
	**Brazil**	10	5228	9443	40(16–64)		**French Caribbean**	3	2372	10610	28(8–48)						
	**Colombia**	2	3208	5472	57(50–64)	**Western Pacific**		**3**	**200**	**4784**	**7(2–12)**						
	**Costa Rica**	2	41	206	20(0–53)		**Malaysia**	1	56	945	6(4–7)						
	**Dominican**	2	1100	1209	77(36–0)		**Singapore**	1	71	3293	2(2–3)						
	**Haiti**	1	108	166	65(58–72)		**Vietnam**	1	73	546	13(11–16)						
	**Mexico**	5	2804	5645	62(43–80)	**Overall**		**44**	**11447**	**51599**	**25(22–29)**						
	**Trinidad**	1	118	125	94(90–98)	**Inapparent** proportion		**16**	**1523**	**4025**	**40(24–56)**						
	**Venezuela**	1	1550	2002	77(76–79)												
	**Suriname**	1	325	400	81(77–85)												
	**French Caribbean**	1	732	783	93(92–95)												
	**Sint Eustatius**	1	184	204	90(86–94)												
**Western Pacific**		**21**	**8964**	**33960**	**27(19–35)**												
	**Cambodia**	1	424	837	51(47–54)												
	China***	4	528	9472	7(2–12)												
	**Hong Kong**	2	108	2785	3(0–6)												
	**Malaysia**	3	459	4218	33(6–61)												
	**Singapore**	6	6427	11718	45(38–52)												
	**Solomon**	1	202	515	39(35–43)												
	**Taiwan**	2	377	2479	15(10–20)												
	**Vietnam**	2	439	1936	25(17–33)												
**Travellers**		**3**	**158**	**1260**	**11(3–19)**												
**Overall**		**102**	**46827**	**124013**	**38 (32–43)**												
**Inapparent**^**#**^ proportion		**24**	**9755**	**13586**	**80(72–88)**												

The region refers to *Eastern Mediterranean Region, ** Southern United States, including Texas, Puerto Rico, Florida, Virgin Islands, and *** Guang Dong province, Southern China. ^#^proportion of inapparent/total infections.

Among regions tested, the top five countries/areas with the highest infection rates were Trinidad (94%, 95% CI: 90–98), French Caribbean (93%, 95% CI: 92–95), Sint Eustatius (90%, 95% CI: 86–94), Suriname (81%, 95% CI: 77–85), and Bangladesh (80%, 95% CI: 78–82); and on the contrary, countries and areas with the lowest DENV infection rates were Croatia (1%, 95% CI: 0–1), HongKong (3%, 95% CI: 0–6), France (3%, 95% CI: 2–4) and Congo and Zambia (both 4%, 95% CI: 3–5) (Table 1).

For subgroup analysis (Table 2), DENV seroprevalences were not significantly different in developed (30%, 95% CI: 15–46) versus developing (38%, 95% CI: 31–44) countries. Urban area had a higher DENV seroprevalence (39%, 95% CI: 30–49) than that of rural areas (23%, 95% CI: 15–32), but did not achieve a statistical significance based on their 95% CIs. Moreover, DENV seroprevalences were similar during the period 2000–2009 (40%, 95% CI: 25–54) and 2010–2019 (37%, 95% CI: 29–44). Globally, there were no statistical differences in DENV seroprevalences when compared adults (44%, 95% CI: 28–59) with children/adolescents (38%, 95% CI: 26–50). The seroprevalence was estimated to be 52% (95% CI: 26–78) in blood donors, 27% (95% CI: 11–44) in pregnant women, and 33% (95% CI: 26–41) in general population for DENV. Prevalence of IgG antibody to DENV (39%, 95% CI: 33–46) was significantly higher than that of the IgM antibody (4%, 95% CI: 3–5). The subgroup analysis for different regions is shown in Tables A, B, C, D, E and F in S2 Text.

**Table 2:** Seroprevalence of DENV infection and subgroup analysis.

Characteristics	African Region (%)	Eastern Mediterranean Region (%)	European Region (%)	South-East Asian Region (%)	Region of the Americas (%)	Western Pacific Region (%)	Overall (%)
**Development stage**							
Developed	/	/	4 (0–7)	/	42 (12–71)	/	30 (15–46)
Developing	12 (8–15)	27 (21–34)	/	56 (39–73)	52 (35–68)	27 (19–35)	38 (31–44)
**Area**							
Urban	11 (5–17)	34 (20–48)	/	65 (55–74)	50 (29–70)	17 (9–24)	39 (30–49)
Rural	14 (12–16)	20 (15–26)	/	33 (21–45)	22 (17–27)	20 (7–33)	23 (15–32)
**Study date**							
2000–2009	15 (11–19)	10 (1–18)	/	27 (0–75)	65 (51–79)	26 (9–42)	40 (25–54)
2010–2019	7 (4–11)	28 (22–33)	4 (0–7)	64 (51–76)	42 (25–59)	28 (16–39)	37 (29–44)
**Age**							
Adults	14 (12–16)	37 (22–52)	/	59 (52–66)	56 (8–100)	40 (19–61)	44 (28–59)
Children	13 (-1-28)	25 (21–29)	/	50 (34–66)	42 (22–63)	30 (5–56)	38 (26–50)
**Population**							
Blood donors	/	/	/	/	/	/	52 (26–78)
Pregnant women	/	/	/	/	/	/	27 (11–44)
General population	13 (7–18)	27 (19–35)	6 (0–12)	56 (14–97)	54 (29–79)	26 (14–37)	33 (26–41)
**Immunoglobulin**							
IgG	13 (8–17)	29 (23–34)	4 (0–7)	59 (47–70)	54 (40–69)	28 (19–36)	39 (33–46)
IgM	4 (0–10)	5 (4–6)	0 (0–1)	4 (1–7)	6 (3–9)	4 (3–5)	4 (3–5)
**Inapparent proportion**	58 (51–66)	19 (17–21)	/	93 (89–98)	79 (70–88)	90 (83–96)	80 (72–88)

There were 24 reports included for analysis of the proportion of inapparent DENV infections among all. Globally, the average proportion of inapparent/total DENV infections was 80% (95% CI: 72–88), ranged from 19% (95% CI: 17–21) in Eastern Mediterranean Region to 93% (95% CI: 89–98) in South-East Asia Region (Table 2 and Table G in S2 Text).

### Seroprevalence of CHIKV infection and subgroup analysis

The analysis for CHIKV seroprevalence included 44 studies involving 51,599 individuals from 29 countries and areas. The overall seroprevalence of CHIKV was 25% (95% CI: 22–29). The prevalence rate of CHIKV infection across the world is shown in Fig 2B. The South-East Asian Region had the highest seroprevalence among all WHO regions (42%, 95% CI: 17–67); In contrast, the Eastern Mediterranean Region had the lowest infection rate (2%, 95% CI: 0–5).

Among regions tested, the top five countries/areas with highest infection rates were Cameroon (68%, 95% CI: 25–100), Comoros (63%, 95% CI: 58–68), Haiti (58%, 95% CI: 56–59), Thailand (49%, 95% CI: 6–92), and Indonesia (39%, 95% CI: 32–45). On the contrary, Turkey (0%, 95% CI 0–1), Gabon (1%, 95% CI: 0–2), Iraq and Croatia (both 1%, 95% CI: 0–1) were ranked those countries with the lowest infection rates (Table 1).

Subgroup analysis (Table 3) showed no significant differences in seroprevalences of CHIKV between developed (14% 95% CI: 6–22) and developing (26%, 95% CI: 22–30) countries, as well as between urban (30%, 95% CI: 4–56) and rural areas (40%, 95% CI: 24–56). Interestingly, there was a declining trend of CHIKV seropositivity over time, as CHIKV seroprevalence was significantly lower during the period 2010–2019 (18%, 95% CI: 14–22) than 2000–2009 (35%, 95% CI: 27–43). However, there was no obvious time trend of CHIKV seroprevalence found in South-East Asian Region. The seroprevalence of CHIKV was estimated to be 18% (95% CI: 9–27) in blood donors, 35% (95% CI: 14–55) in pregnant women, and 29% (95% CI: 23–35) in the general population. For CHIKV, the seroprevalence of IgM and IgG antibodies was 17% (95% CI: 12–23) and 24% (95% CI: 21–28), respectively, which were not significantly different. The subgroup analysis for different regions is shown in Tables A, B, C, D and E in S3 Text.

**Table 3:** Seroprevalence of CHIKV infection and subgroup analysis.

Characteristics	African Region(%)	Eastern Mediterranean Region(%)	European Region(%)	South-East Asian Region(%)	Region of the Americas(%)	Western Pacific Region(%)	Overall(%)
**Development stage**							
Developed	/	/	5 (0–14)	/	19 (0–39)	/	14 (6–22)
Developing	33 (24–41)	2 (0–5)	0 (0–1)	42 (17–67)	25 (10–40)	7 (2–12)	26 (22–30)
**Area**							
Urban	/	/	/	/	/	/	30 (4–56)
Rural	/	/	/	/	/	/	40 (24–56)
**Study date**							
2000–2009	39 (27–51)	/	/	37 (0–82)	/	6 (4–7)	35 (27–43)
2010–2019	19 (6–32)	2 (0–5)	1 (0–1)	35 (19–51)	23 (13–34)	8 (0–19)	18 (14–22)
**Population**							
Blood donors	/	/	/	/	/	/	18 (9–27)
Pregnant women	/	/	/	/	/	/	35 (14–55)
General population	41 (29–54)	2 (0–5)	/	36 (0–100)	28(8–49)	2 (2–3)	29 (23–35)
**Immunoglobulin**							
IgG	30 (22–37)	2 (0–5)	3 (1–5)	49 (33–66)	24 (12–36)	7 (2–12)	24 (21–28)
IgM	24 (15–33)	/	/	16 (0–38)	8 (0–15)	/	17 (12–23)
**Inapparent proportion**	26 (17–35)	/	/	53 (0–100)	48 (35–61)	/	40 (24–56)

By analyzing 16 studies, the average proportion of inapparent CHIKV infections to all CHIKV infections was 40% (95% CI: 24–56), ranged from 18% (95% CI: 5–31) in European Region to 53% (95% CI: 0–100) in South-East Asia Region (Table 3 and Table F in S3 Text).

### Seroprevalence of ZIKV infection

A total of 14 studies involving 18,394 participants from 12 countries and areas were included for analysis of ZIKV seroprevalence (Table 1). The pooled prevalence of ZIKV infection was 18% (95% CI: 12–25) across the world. The prevalence rate of ZIKV infection across the world is shown in Fig 2C. Region of the Americas had the highest seroprevalence among all WHO regions (34%, 95% CI: 24–45); while African Region had the lowest infection rate (4%, 95% CI: 2–7). There were no reports from South-East Asian Region and European Region. The average proportion of inapparent ZIKV infections to all ZIKV infections was 50% (95% CI: 18–82) (Table 1).

### Co-infection between DENV, CHIKV and ZIKV

Of total 133 articles, 7 studies analyzed the co-infection of DENV, CHIKV and ZIKV, including 5 for DENV/CHIKV, 1 for DENV/ZIKV, and 1 for both DENV/CHIKV and CHIKV/ZIKV. The co-prevalence between DENV and CHIKV varied greatly from near 0% to 41% and the pooled rate was 11% (95% CI: 5–17). The high co-infection rates were observed in South-East Asian Region (India and Thailand), indicated the overlapping endemicity of CHIKV and DENV. Though limited evidence on co-infections of DENV/ZIKV and CHIKV/ZIKV, the low co-infection rates can be seen from one report as 2% (95% CI: 1–3) and almost 0% (95% CI: 0–1), respectively (Table 4 and Table A and Fig A in S4 Text).

**Table 4:** Analysis for co-infections of DENV, CHIKV and ZIKV.

	DENV/CHIKV	CHIKV/ZIKV	DENV/ZIKV
**Number of reports**	6	1	1
**Number of cases**	557	1	17
**Number of samples**	3727	367	978
**Seroprevalence (%, 95%CI)**	11(5–17)	0 (0–1)	2 (1–3)

### The detection of nucleic acids

Of total 133 publications, both nucleic acids and antibodies were detected in 6 studies, including 2 for ZIKV, 2 for DENV, 1 for CHIKV and 1 for all three viruses. None of the individuals were found to be DENV or CHIKV viremic by screening nucleic acids. ZIKV RNA were detected in 2 studies; however the positive rates tested by nucleic acid detection method were lower than those tested by antibody detection method (6.7% vs.14.0%; 7.4% vs. 21.5%), respectively (Table B in S4 Text).

## Discussion

In this study, we estimated the global, regional, and national seroprevalences of three mosquito-borne diseases (DENV, CHIKV and ZIKV) using 133 published articles. The results indicated that DENV and CHIKV have spread throughout the world; and the pooled seroprevalences of DENV, CHIKV, and ZIKV were 38%, 25%, and 18% respectively, revealing a heavy burden of the diseases to the world population and especially in specific countries/regions. We also generated extensive information about the profile of seroprevalence in different subgroups and found no significant differences between some subgroups.

There were heterogeneous results across regions, with the highest seroprevalence in South-East Asian Region for DENV and CHIKV, and Regions of the Americas for ZIKV. As the Global Burden of Disease study estimated [5], the disease caused by DENV accounted for 52% of the disease burden in South-East Asia Region. CHIKV has been endemic in Regions of South-East Asia and Sub-Saharan African, where two cycles of enzootic sylvan transmission and urban human-mosquito-human transmission exist [27]. It was estimated that there were 47 (out of 55) countries/areas in the Regions of the Americas confirmed endemic ZIKV circulation in 2017, infecting about 100 million people [28]. Regarding the trending overtime, a progressively growing DENV seroprevalence has been observed since 2000, indicating a continuous dengue outbreak around the world. Similarly, there was no obvious time trend of CHIKV seroprevalence found in South-East Asian Region, where CHIKV is most prevalent. However, the seroprevalence was declined in the period of 2010–2019 as compared with the period of 2000–2009 in African Region. Further studies are needed to investigate the specific reasons for the decrease of CHIKV pooled seroprevalence in the past ten years, to find any clues about the prevention and control of mosquito-borne diseases.

Subgroup analyses regarding the seroepidemiological profile of DENV and CHIKV were conducted in the present study. Mosquito-borne diseases are generally considered to be more prevalent in developing countries [29]. In this study, the seroprevalences of DENV and CHIKV in developing countries were higher than those in developed countries in Region of the Americas, but the differences did not reach a statistical significance. There is a possibility of explaining this. Besides meteorological factors, socioeconomic status is a factor that affects the transmission of mosquito-borne diseases. Most of countries in endemic regions were defined as developing countries with high population density but low socioeconomic status [30,31]. The prevalence of these diseases may be underestimated due to limited laboratory facilities and inadequate testing capacity. It was exactly opposite to that of low epidemic regions, where countries were relatively developed. In our analysis, the seroprevalences of DENV in urban areas were higher than those in rural areas in South-East Asian Region and Region of the Americas. However, there was no difference between rural and urban areas for the overall seroprevalence of DENV and CHIKV. These mosquito-borne diseases are considered more widespread in urban areas because of favorable habitats for vector mosquitoes in urban environments [32]. However, a seroepidemiological study on DENV demonstrated substantial underdiagnosis and underreporting of dengue cases, due to the inability of the primary medical care system in rural areas [33]. Nowadays, with rapid urbanization and accelerated population mobility, the gap between urban and rural areas has narrowed [34].

A variety of diagnostic methods are available for serological tests of mosquito-borne diseases, with ELISA IgG/IgM most commonly used. ELISA IgG is recommended due to its higher sensitivity, stronger response and longer lasting time of IgG [20]. However, it is less specific for cross-reactivity between antibodies against different arboviruses [20]. In our study, dual seropositivity of CHIKV and DENV was reported in the South-East Asia Region, African Region, and Region of the Americas. Besides the possibility of cross-reactivity, CHIKV/DENV dual seropositivity may indicate overlapping endemicity between CHIKV and DENV in these geographic regions. Moreover, prevalence of IgG antibody to DENV (38%) was significantly higher than that of IgM antibody (4%). The activity of IgM represents recent infection, as it appears during the acute phase of the infection, probably 5 days after fever onset, and it can only persist a few days; while IgG occurs after viremia phase and can last for a long time, even lifelong, whose existence demonstrates a past infection [35,36]. That may explain a higher seroprevalence of IgG than IgM.

A considerable proportion of DENV, CHIKV, and ZIKV infections remain inapparent, especially DENV in our study with a percentage up to 80%, which is consistent with the report of a review, in which inapparent DENV infection estimated to be 70–91% of the total [37]. Although a clear definition of inapparent infection is lacking, here in this meta-analysis it includes “subclinical infection” referring to infection but without major symptoms as to require medical care, and “asymptomatic infection” which means complete absence of any relevant symptoms. At present, the mechanisms for inapparent infection remain elusive, involving complex interactions between host, vector, and viruses [38]. There are a few of reports depicting potential epidemiological factors for inapparent DENV infection, including the age of host when infected, the time interval between consecutive infection, previous DENV infecting serotype, and the concentrations of preexisting heterotypic neutralizing antibodies [39–41]. Although no evidence suggests a direct impact of inapparent infection on patient health, it may increase the risk of severe outcomes in the context of secondary DENV infection with different serotypes or another flavivirus [42]. On the other hand, despite their lower average level of viremia, asymptomatic people can be infectious to mosquitoes [43]. Without detectable symptoms, DENV viremic individuals, through their undisrupted daily routines, have the potential to contribute significantly more to virus transmission to mosquitoes than sick people did. Indeed, a recent model analysis suggests that inapparent infections may account for 84% of DENV transmission [44]. The high proportions of inapparent infections in our study indicate that efforts should be scaled up to develop a more effective surveillance and monitoring system. To be noted, the studies included for analysis of inapparent infection were mainly retrospective, which is prone to introduce bias. Confirmation of these results needs prospective surveys to provide more strict estimates.

It is the first time to depict a global blueprint regarding seroepidemiology of three important arbovirus infections with a large sample size and detailed subgroup analysis. However, there are still several limitations. Firstly, the scope of literature searching was restricted to title, and possibly there were some potential reports missed. Besides, this is a systematic review and meta-analysis of the published studies to estimate the pooled prevalence of DENV, CHIKV and ZIKV Infection; however, in some countries data on the prevalence are not available. Secondly, we identified a significant heterogeneity among the studies which might be on account of a series of factors, such as date of study, socioeconomic status, demographic characteristics, diagnostic tests, and cut-offs adopted. The heterogeneity of single-rate meta-analysis is usually higher than the two-group study, for instance, case-control study and randomized controlled trials, because the data were extracted only from one group. Thus, *I*^*2*^ statistic often exceeds 90% in meta-analysis studies [26,45]. In addition, the quality and the accuracy of studies from which the data have been gathered cannot be guaranteed. Finally, in most of the studies included for analysis, only immunoglobulin was detected but not confirmed by nucleic acid detection test or neutralization test which is the “gold standard” for diagnosis of arbovirus diseases.

In conclusion, this study provides evidence that arboviruses DENV, CHIKV and ZIKV have spread worldwide, and DENV, and CHIKV co-circulate in some geographical regions. Considering high proportions of inapparent infections, surveillance of only symptomatic cases is insufficient to evaluate the persistence of infection. A considerable proportion of cases are undiagnosed or unreported and the true burden of these arbovirus infections is largely unknown, which reinforces the need for population screening especially in epidemic areas.

## Supporting information

S1 Text Characteristics of studies included in the systematic review and meta-analysis. Table A. Characteristics of studies included in the systematic review and meta-analysis for DENV. Table B. Characteristics of studies included in the systematic review and meta-analysis for CHIKV. Table C. Characteristics of studies included in the systematic review and meta-analysis for ZIKA.
(DOCX)

S2 Text Subgroup analysis of DENV seroprevalence. Table A. Seroprevalence of DENV infection for developing and developed countries. Table B. Seroprevalence of DENV infection for urban and rural areas. Table C. Time trend of DENV seroprevalence. Table D: Seroprevalence of DENV infection in general population. Table E. Seroprevalence of DENV infection stratified by diagnostic tests. Table F. Seroprevalence of DENV infection stratified by age. Table G. Proportion of DENV inapparent infection.
(DOCX)

S3 Text Subgroup analysis of CHIKV seroprevalence. Table A. Seroprevalence of CHIKV infection for developing and developed countries. Table B. Time trend of CHIKV seroprevalence. Table C. Seroprevalence of CHIKV infection in general population. Table D. Seroprevalence of CHIKV infection stratified by diagnostic tests. Table E. Seroprevalence of CHIKV infection for urban and rural areas. Table F. Proportion of CHIKV inapparent infection.
(DOCX)

S4 Text Information of cross-infection and nucleic acid detection. Table A. The publications reporting cross-infection between any two of three arboviruses. Table B. The publications reporting both results of nucleic acid and antibody. Fig A. Forest plot of the pooled seroprevalence of cross-infection.
(DOCX)

S5 Text Assessments of the quality of the studies. Table A. Assessments of the quality of the studies.
(DOC)

S6 Text PRISMA 2009 checklist. Table A. PRISMA 2009 checklist.
(DOC)
