# Supplementary material for: The worldwide seroprevalence of DENV, CHIKV and ZIKV infection: A systematic review and meta-analysis
Source: PLoS Negl Trop Dis. 2021 Apr 28;15(4):e0009337. doi: 10.1371/journal.pntd.0009337 (PMC8109817; doi:10.1371/journal.pntd.0009337)
Supplement: S1 Text — Table A. Characteristics of studies included in the systematic review and meta-analysis for DENV. Table B. Characteristics of studies included in the systematic review and meta-analysis for CHIKV. Table C. Characteristics of studies included in the systematic review and meta-analysis for ZIKA. (DOCX) [file pntd.0009337.s001.docx]

S1 Appendix

Tables A, B and C: Characteristics of studies included in the systematic review and meta-analysis (page 2 – 16) .

References (page 16 – 26).

Table A: Characteristics of studies included in the systematic review and meta-analysis for DENV.

| **First author (Publication Year)** | **Country** | **Rural or Urban** | **WHO Rigion** | **Study Period** | **Population type** | **Samplingmethod** | **Recruitment** | **Laboratory test** | **Age range** | **Male proportion (%)** | **DENV Prevalence (%)** | **DENV Inapparent proportion (%)** | **Bias quality** |
| --- | --- | --- | --- | --- | --- | --- | --- | --- | --- | --- | --- | --- | --- |
| Al-Azraqi(2013) | Saudi Arabia | NM | Eastern Mediterranean | NM | General population | simple random | Hospital | ELISA-IgG | 2–92 | 69.9 | 306/965(31.7 ) | NM | M |
| Al-Raddadi(2019) | Saudi Arabia | NM | Eastern Mediterranean | 2016-2017 | General population | multistage | Population | ELISA-IgG | all ages | 60.4 | 1710/6397(26.7 ) | 324/1710(81.1) | M |
| Amaya-Larios(2018) | Mexico | NM | the Americas | 2016-2016 | School children | multistage | School | ELISA-IgG | 6–17 | 48.4 | 715/2134(33.5 ) | NM | M |
| Ang(2015) | Singapore | Urban | Western Pacific | 2010 | General population | multistage | Population | ELISA-IgG | 18–79 | 43·1 | 1870/3293(56.8 ) | NM | M |
| Argolo(2013) | Brazil | NM | the Americas | 2009-2010 | Pregnant women | NM | Hospital | ELISA-IgG,IgM | NM | 0 | 272/505( 53.9) | 184/272(67.6) | M |
| Ashshi(2017) | Saudi Arabia | Urban | Eastern Mediterranean | 2015-2016 | Blood Donors | simple random | Blood donor center | ELISA-IgG,IgM | 25- 55 | 100 | 367/910(40.3) | NM | M |
| Auerswald(2019) | Maderia | NM | European | 2015 | General population | convenience | Hospital, Laboratory | ELISA-IgG | 11-92 | 38.0 | 32/358(8.9) | NM | M |
| Braga(2010) | Brazil | Urban | the Americas | 2005-2006 | General population | cluster | Household | ELISA-IgG | 5 - 64 | 43.1 | 2380/2819(84.4) | NM | M |
| Brunkard(2007) | America | Urban | the Americas | 2004-2014 | General population | multistage | Household | ELISA-IgG,IgM | 15–88 | 53.5 | 120/300(40.0) | NM | H |
| Brunkard(2007) | Mexico | Urban | the Americas | 2004-2014 | General population | multistage | Household | ELISA-IgG,IgM |  |  | 234/300(78.0) | NM | H |
| Campbell(2007) | Trinidad | NM | the Americas | 2003-2004 | Neonates | convenience | Hospital | ELISA-IgG,IgM | 0 | NM | 118/125(94.4) | NM | M |
| Carabali(2017) | America | Urban | the Americas | 2011-2014 | General population | multistage | Population | ELISA-IgG | 1-65 | 33.5 | 2246/3684(61.0 ) | 2173/2246(96.7) | H |
| Chiaravall (2019) | Brazil | Urban | the Americas | 2015-2016 | General population | cluster | Household | ELISA-IgG | 10-91 | 39.4 | 986 /1322(74.6 ) | 566/986(57.4 ) | H |
| Chien(2019) | Taiwan,China | NM | Western Pacific | 2015-2015 | General population | convenience | Center recruitment | ELISA-IgG,IgM | 20–88 | 42.1 | 242/1391(17.4) | 206/242((85.1) | M |
| Chow(2005) | Singapore | NM | Western Pacific | 1998-2000 | Others | convenience | School | ELISA-IgG | 19-26 | 53.8 | 41/184(22.0) | NM | M |
| Darcy(2001) | Solomon | NM | Western Pacific | 1994-1995 | Blood Donors | NM | Blood donor center | ELISA-IgG | NM | NM | 202/515(39.0) | NM | M |
| de Almeida(2018) | Brazil | Urban | the Americas | 2017 | Blood Donors | convenience | Blood donor center | ELISA-IgG,IgM | 18-69 | 65.4 | 5/298(1.7) | NM | M |
| Dhanoa(2018) | Malaysia | Both | Western Pacific | 2015 | General population | NM | Household | ELISA-IgG,IgM | ≥18 | 41.9 | 240/277 (86.6) | 209/240(87.1) | M |
| Dhar-Chowdhury(2017) | Bangladesh | Urban | South-East Asia Region | 2012 | General population | multistage | Household | ELISA-IgG,IgM | 1-77 | 43.3 | 900/1125(80.0) | NM | M |
| Eldigail(2018) | Sudan | Urban | Eastern Mediterranean | 2016-2017 | General population | multistage | Household | ELISA-IgG | ≥18 | 59.8 | 334 /701 (47.6) | NM | M |
| Ellis(2015) | Kenya | Urban | African | 2013 | General population | cluster | Household | ELISA-IgM，qRT-PCR | 0.1–94 | 40.3 | 210/1500(14) | 117/210(56.0) | M |
| Fox-Lewis(2019) | Cambodia | Urban | Western Pacific | 2017 | Age-specific subgroups | simple random | Hospital | ELISA-IgG | 0-15 | 50.0 | 424/837(50.7) |  | M |
| Gabor(2016) | Gabon | NM | African | 2007 | Infants | NM | Previous studies | ELISA-IgG | 2.5 | 49.0 | 20/162(12.3) | NM | M |
| Garg(2017) | India | Both | South-East Asia Region | 2011-2012 | Age-specific subgroups | simple random | School | ELISA-IgG | 5-10 | 47.4 | 1525/2558(59.6) | NM | H |
| Guo(2014) | China | Urban | Western Pacific | 2003-2011 | General population | simple random | Hospital | ELISA-IgG | NM | NM | 136/5586(2.4) | NM | M |
| Hono´ rio(2009) | Brazil | Both | the Americas | 2008 | General population | simple random | Household | ELISA-IgG、IgM | 1-79 | NM | 175/258(67.8) | 16/24(66.7) | M |
| Humphrey(2019) | Qatari | NM | Eastern Mediterranean | 2013-2016 | General population | simple random | Blood donor center | ELISA-IgG | ≥18 | 100 | 473/1992(23.7) | NM | M |
| Iturrino-Monge(2006) | Costa Rican | NM | the Americas | 2002-2003 | Age-specific subgroups | simple random | Hospital | ELISA-IgG | 1- 11 | 73.0 | 38/103(36.9) | NM | H |
| Iturrino-Monge(2006) | Costa Rican | NM | the Americas | 2002-2003 | Age-specific subgroups | simple random | Hospital | ELISA-IgG | 1- 11 | 73.0 | 3/103(2.9) | NM | H |
| Jamjoom(2016) | Saudi Arabia | Urban | Eastern Mediterranean | NM | General population | simple random | Center recruitment | ELISA-IgG | 3-80 | 53.0 | 927/1939(47.8) | NM | M |
| Jamjoom(2016) | Saudi Arabia | NM | Eastern Mediterranean | NM | Blood Donors | simple random | Blood donor center | ELISA-IgG | NM | NM | 68/184(37.0) | NM | M |
| Jeewandara(2015) | Sri Lanka | Urban | South-East Asia Region | 2013-2014 | General population | simple random | Center recruitment | ELISA-IgG | all ages | 49.1 | 1152/1689(68.2) | 1019(88.5) | H |
| Jing(2019) | China | Both | Western Pacific | 2015 | General population | multistage | Population | ELISA-IgG | 1-84 | 46.2 | 56/850(6.6) | NM | H |
| Kuniholm(2006) | Cameroon | Rural | African | 2002-2003 | General population | simple random | Population | PRNT | >16 | 55.9 | 32/256(12.5) | NM | M |
| Laoprasopwattana(2016) | Thailand | NM | South-East Asia Region | 2009-2010 | Pregnant women | simple random | Hospital | IFA,ELISA-IgG,IgM | >15 | 0 | 288/319(90.3) | 286/288(99.3) | M |
| L’Azou(2015) | French Caribbean islands | NM | the Americas | 2011 | Blood Donors | simple random | Blood donor center | ELISA-IgG | 18-70 | 47.0 | 732/783(93.5) | NM | H |
| Leder(2013) | travellers | NM | / | 1998-2000 | General population | simple random | Population | ELISA-IgG | NM | 49.1 | 25/282(8.9) | NM | M |
| Lee(2018) | HongKong, | NM | Western Pacific | 2013-2015 | General population | simple random | Hospital | ELISA-IgG | all ages | NM | 97/2100(4.6) | NM | M |
| Leslie(2014) | Sint Eustatius | NM | the Americas | 2011 | General population | simple random | School | ELISA-IgG | >10 | 26.5 | 184/204(90.2) | NM | H |
| Liu(2018) | China | Urban | Western Pacific | 2013-2015 | General population | simple random | Population | ELISA-IgG, IgM | all ages | 36.6 | 295/2085(14.2) | 235/243(96.7) | H |
| Liu(2019) | China | Urban | Western Pacific | 2016 | Pregnant women | simple random | Hospital | ELISA-IgG, IgM | 18–44 | 0 | 41/951(4.3) | 41/41(100) | H |
| Lo(2013) | HongKong, | Urban | Western Pacific | 2007-2009 | General population | simple random | Center recruitment | ELISA-IgG | >16 | 49.8 | 11/685(1.6) | NM | M |
| Low(2015) | Singapore | NM | Western Pacific | 2009-2010 | Blood Donors | stratified | Blood donor center | ELISA-IgG,IgM | 16–60 | 48.3 | 1885/3627(52.0) | NM | H |
| Lozier(2018) | America | Urban | the Americas | 2016 | General population | convenience | Household | ELISA-IgM,RT-PCR, | all ages | 37.3 | 3/367(0.8) | NM | M |
| Mai(2018) | Vietnam | Both | Western Pacific | 2011 | General population | multistage | Household | ELISA-IgG, IgM | 3–60 | 48.5 | 308/1485(20.7) | NM | M |
| Malavige(2006) | Sri Lanka | Urban | South-East Asia Region | NM | School children | simple random | School | ELISA-IgG | 6-18 | 49.8 | 107/313(34.2) | 107/107(100) | L |
| Martins(2014) | Brazil | Urban | the Americas | 2010 | Age-specific subgroups | simple random | Population | ELISA-IgG | 6 months-12 | 51.3 | 12/411(2.9) | 11/12(91.7) | H |
| Mazaba-Liwewe(2014) | Zambia | NM | African | 2010 | General population | multistage | Household | ELISA-IgG | >9 months | 46.7 | 149/3624(4.1) | NM | M |
| Mease(2011) | Kenya | Rural | African | 2004 | General population | multistage | Population | ELISA-IgG | >18 | 35.9 | 162/1141(14.4) | NM | M |
| Messenger(2014) | America | NM | the Americas | 2012 | General population | convenience | Population | ELISA-IgG,IgM | 18-84 | 52.4 | 18/173(10.4) | NM | H |
| Mishra(2018) | India | Urban | South-East Asia Region | 2017 | General population | multistage | Center recruitment | ELISA-IgG | 1month-85 | 50.4 | 1163/1434(81.1) | 1113/1205(92.4) | M |
| Mohammed(2012) | America | NM | the Americas | 2006 | Blood Donors | simple random | Blood donor center | ELISA-IgG | 18-80 | 84.0 | 275/300(92.0) | NM | H |
| Mohsin(2016) | Pakistan | NM | Eastern Mediterranean | NM | Age-specific subgroups | multistage | Household | ELISA-IgG | 1-12 | 60.3 | 100/400(25.0) | NM | M |
| Murhekar(2019) | India | Both | South-East Asia Region | 2017-2018 | Age-specific subgroups | multistage | Household | ELISA-IgG | 5-45 | 47.3 | 5338/12300(43.4) | NM | H |
| Nujum(2018) | India | NM | South-East Asia Region | NM | Pregnant women | NM | Hospital | ELISA-IgG | NM | 0 | 7/143(6.9) | 7/7(100) | L |
| Obaidat(2018) | Jordan | Both | Eastern Mediterranean | 2015-2016 | General population | simple random | Hospital | ELISA-IgG | all ages | 38.7 | 219/892(24.6) | NM | M |
| Ochieng(2015) | Kenya | Both | African | 2007 | General population | multistage | Household | ELISA-IgG | 15-64 | 39.4 | 143/1091(13.1) | NM | M |
| Overbosch(2014) | Suriname | NM | the Americas | 2008-2011 | Others | convenience | Center recruitment | ELISA-IgG | 18-89 | 37.0 | 325/400(81.3) | NM | H |
| Pacsa(2003) | Kuwaiti | NM | Eastern Mediterranean | 1997-1999 | Others | NM | Hospital | ELISA-IgG, IgM | all ages | NM | 70/499(14.0) | NM | M |
| Padbidri(2002) | India | NM | South-East Asia Region | 1988-1989 | General population | simple random | Not specified | HI | all ages | 56.5 | 75/2401(3.1) | NM | M |
| Pavı´a(2018) | Mexico | Urban | the Americas | 2014-2016 | Age-specific subgroups | simple random | School | ELISA-IgG | 0-15 | 51.3 | 862/1844(46.8) | NM | H |
| Pavı´a(2018) | Mexico | Urban | the Americas | 2014 | Others | stratified | Not specified | ELISA-IgG | all ages | 37.9 | 1227/1667(73.6) | 1163/1227(94.8) | H |
| Pem(2015) | Croatia | NM | European | 2011-2012 | Others | NM | Hospital | ELISA-IgG | 2-87 | 44.5 | 7/1180(0.6) | NM | M |
| Pereira(2018) | Brazil | NM | the Americas | 2013-2014 | Others | NM | Not specified | ELISA-IgG,IgM | NM | 35.1 | 263/1560(16.9) | NM | M |
| Piedrahita(2018) | America | Urban | the Americas | 2010 | School children | NM | Previous studies | ELISA-IgG | 5-19 | 48.5 | 962/1788(53.8) | NM | H |
| Prayitno(2017) | Indonesia | Urban | South-East Asia Region | 2014 | Age-specific subgroups | cluster | Household | ELISA-IgG | 1-18 | 47.8 | 2216/3194(69.4) | 1943/2216(87.7) | H |
| Quyen(2018) | Vietnam | Rural | Western Pacific | 2015 | Age-specific subgroups | multistage | Population | ELISA-IgG | 1-10 | NM | 131/451(29.0) | NM | M |
| Ramos(2008) | America | Urban | the Americas | 2005 | General population | multistage | Household | ELISA-IgG,IgM | >5 | 41.8 | 49/141(34.8) | NM | H |
| Ramos(2008) | Mexico | Urban | the Americas | 2005 | General population | multistage | Household | ELISA-IgG,IgM |  |  | 101/132(76.5) | NM | H |
| Ranjan(2016) | India | Both | South-East Asia Region | 2012 | Blood Donors | NM |  | ELISA-IgG,IgM，RT-PCR | 19-51 | 97.0 | 118/200(59.0) | NM | M |
| Ratnam(2012) | travellers | NM | / | 2007-2010 | Others | NM | Center recruitment | ELISA-IgG | 17-78 | 59.4 | 20/387(5.2) | 20/20(100) | H |
| Rioth(2011) | Haiti | Urban | the Americas | 2007 | Others | NM | Center recruitment | PRNT | 7-36 months | 54.2 | 108/166(65.0) | NM | M |
| Sacramento(2018) | Brazil | Rural | the Americas | 2015 | General population | census | Not specified | ELISA-IgG | all ages | 41.4 | 62/280(22.1) | NM | M |
| Sanchez(2013) | travellers | NM | / | 2008-2009 | Others | NM | Center recruitment | ELISA-IgG | 11-86 | 46.8 | 113/591(19.0) | 60/71(84.5) | H |
| Schwarz(2012) | Madagascar | Urban | African | 2010 | Pregnant women | NM | Center recruitment | IFA-IgG, IgM | 12-50 | 0 | 97/1244(7.8) | 39/61(63.9) | M |
| Shah(2019) | India | Both | South-East Asia Region | 2016 | Age-specific subgroups | simple random | Not specified | ELISA-IgG | 5-15 | 50.7 | 168/819(20.5) | NM | H |
| Shah(2017) | India | Rural | South-East Asia Region | 2011 | General population | stratified | Not specified | ELISA-IgG | all ages | NM | 390/855(45.6) | NM | H |
| Shaukat(2018) | Pakistan | Both | Eastern Mediterranean | 2017 | General population | simple random | Hospital | ELISA-IgG | NM | 56.7 | 69/240(28.8) | NM | H |
| Sissoko(2010) | Mayotte | NM | African | 2006 | General population | multistage | Household | ELISA-IgG | >2 | 43.2 | 262/1154(22.7) | NM | H |
| Slavov(2019) | Brazil | NM | the Americas | 2015-2016 | Blood Donors | NM | Blood donor center | ELISA-IgM, PCR | NM | 65.8 | 32/475(6.7) | NM | M |
| Soghaier(2015) | Sudan | Urban | African | 2011 | General population | multistage | Household | ELISA-IgG | 13-85 | 40.0 | 46/491(9.4) | NM | H |
| Soghaier(2014) | Sudan | NM | African | 2012 | General population | multistage | Household | ELISA-IgG | 15-60 | 49.0 | 166/600(27.7) | NM | H |
| Succo(2018) | France | NM | European | 2015 | General population | NM | Household | ELISA-IgG, IgM | 2-86 | 44.4 | 17/562 (3.0) | NM | M |
| Tan(2008) | Malaysia | Urban | Western Pacific | 2006 | Pregnant women | NM | Center recruitment | ELISA-IgM | NM | 0 | 63/2531(2.5) | 56/63(88.9) | H |
| Thisyakorn(2016) | Thailand | NM | South-East Asia Region | NM | Age-specific subgroups | NM | Center recruitment | PRNT | <15 | 56.0 | 65/100(65.0) | NM | M |
| Tiong(2015) | Malaysia | Both | Western Pacific | 2008-2009 | School children | convenience | School | ELISA-IgG | 7-18 | NM | 156/1410(11.1) | NM | M |
| Tissera(2010) | Sri Lanka | NM | South-East Asia Region | 2008 | Age-specific subgroups | simple random | Household | ELISA-IgG | <12 | NM | 412/797(51.7) | NM | M |
| Tsai(2018) | Taiwan | Urban | Western Pacific | 2015-2017 | General population | NM | Center recruitment | ELISA-IgG | 9-85 | 36.4 | 135/1088(12.4) | NM | H |
| Velasco(2014) | Venezuela | NM | the Americas | 2010-2011 | Age-specific subgroups | NM | Household | HI | 5-30 | 43.2 | 1550/2002(77.4) | 1190/1544(77.1) | H |
| Villarroel(2018) | Bolivia | NM | the Americas | 2016-2017 | Blood Donors | NM | Blood donor center | ELISA-IgG | NM | NM | 223/449(49.7) | NM | H |
| Vongpunsawad(2017) | Thailand | NM | South-East Asia Region | 2014 | Others | simple random | Hospital | ELISA-IgG | 0.5-60 | 47.8 | 661/835(79.2) | NM | H |
| Wilder(2004) | Singapore | NM | Western Pacific | 2002 | Others | NM | Hospital | ELISA-IgG | 18-45 | 37.0 | 133/298(44.6) | NM | H |
| Wilder(2005) | Singapore | NM | Western Pacific | NM | Others | NM | Hospital | ELISA-IgG, PRNT | 18-30 | 30.0 | 49/164(29.9) | NM | M |
| Willcox(2018) | Congo | Both | African | 2013-2014 | Age-specific subgroups | simple random | Household | ELISA-IgG | 0.5-5 | NM | 37/978(3.8) | NM | M |
| Yamashiro(2004) | Dominican | NM | the Americas | 2002 | Blood Donors | NM | Blood donor center | ELISA-IgG | 17-79 | 94.6 | 987/1008(97.9) | NM | M |
| Yamashiro(2004) | Dominican | NM | the Americas | 2002 | Age-specific subgroups | NM | Hospital | ELISA-IgG | 3 months -10 | 58.7 | 113/201(56.2) | NM | M |
| Yik(2009) | Singapore | NM | Western Pacific | 2004 | General population | multistage | Previous studies | ELISA-IgG, IgM | 18-74 | 49.6 | 2449/4152(59.0) | NM | H |
| Andayi(2014) | Djibouti | NM | Eastern Mediterranean | 2010-2011 | General population | NM | Household | ELISA-IgG | all ages | 45.4 | 199/911(21.8) | NM | M |
| Rodriguez-Barraquer(2015) | India | NM | South-East Asia Region | 2011 | Age-specific subgroups | cluster | Household | ELISA-IgG | 5-40 | 45.0 | 744/800(93.0) | 737/744(99.1) | H |
| Seidahmed(2012) | Sudan | NM | African | 2008-2009 | General population | stratified | Household | ELISA-IgM | 3 months -80 | 36.0 | 41/791(5.2) | NM | H |
| Teixeira(2002) | Brazil | Urban | the Americas | 1998 | General population | NM | Household | ELISA-IgG | all ages | 42.1 | 1041/1515(68.7) | NM | H |
| Moss(2018) | Mali | NM | African | NM | School children | stratified | School | MBA-IgG | 4-17 | NM | 196/805(24.4) | NM | M |

Table B: Characteristics of studies included in the systematic review and meta-analysis for CHIKV.

| **First author(Publication Year)** | **Country** | **Rural or Urban** | **WHO Rigion** | **Study Period** | **Population type** | **Sample method** | **Recruitment** | **Laboratory test** | **Age range** | **Male proportion (%)** | **CHIKV** | | **Bias quality** |
| --- | --- | --- | --- | --- | --- | --- | --- | --- | --- | --- | --- | --- | --- |
|  |  |  |  |  |  |  |  |  |  |  | **Prevalence (%)** | **Inapparent proportion (%)** |  |
| Ang(2017) | Singapore | Urban | Western Pacific | 2010 | General population | multistage | Population | ELISA-IgG, PRNT | 18–79 | 43·1 | 71/3293(2.2) | NM | M |
| Azami(2013) | Malaysia | Both | Western Pacific | 2009 | Age-specific subgroups | simple random | Not specified | ELISA-IgG | 35–74 | 39.8 | 56/945 (5.9) | NM | M |
| Bacci(2015) | Benin | Urban | African | 2006-2007 | Pregnant women | NM | Hospital | ELISA-IgG,IgM | 14-42 | 0 | 133/352(37.8) | NM | M |
| Barakat(2016) | Iraq | NM | Eastern Mediterranean | 2012-2013 | General population | convenience | Not specified | IFA,MNT | 10–82 | 59.0 | 2/399 (0.5) | NM | M |
| Cortes-Escamilla(2018) | Mexico | NM | the Americas | 2016-2016 | General population | multistage | Household | ELISA-IgG | 2-65 | 25.1 | 114/387 (29.5) | 36/114 (31.6) | M |
| Cunha(2017) | Brazil | Rural | the Americas | 2016 | General population | cluster | Household | ELISA-IgG,IgM | all ages | 48.3 | 24/120(20.0) | 11/24(45.8) | M |
| Demanou(2010) | Cameroon | Rural | African | 2006 | General population | convenience | Not specified | ELISA-IgG,IgM | 8-81 | 30.5 | 95 /105(90.5) | NM | M |
| Dias(2018) | Brazil | Urban | the Americas | 2015 | General population | census | Household | ELISA-IgG,IgM | ≥1 | 30.8 | 424/831(51.0) | 268/424(62.9) | H |
| Gabor(2016) | Gabon | NM | African | 2007 | Infants | NM | Previous studies | ELISA-IgG | 2.5 | 49.0 | 1/162(0.6) | NM | M |
| Gay(2016) | French Caribbean islands | NM | the Americas | 2014 | General population | convenience | Laboratory | ELISA-IgG,IgM | >6months | 36.5 | 42/203(20.7) | 17/42(40.5) | M |
| Gerardin(2008) | La Réunion Island | NM | African | 2006 | Pregnant women | convenience | Laboratory | ELISA-IgG,IgM | NM | 0 | 162/888(18.2) |  | M |
| Gerardin(2008) | La Réunion Island | NM | African | 2006 | General population | stratified | Household | ELISA-IgG | NM | NM | 967/2442(39.6) | 162/967(16.7) | M |
| Hennessey(2018) | America | NM | the Americas | 2014-2015 | General population | multistage | Household | ELISA-IgG、PRNT | >6months | 44.0 | 171/509(33.6) | 50/171(29.2) | H |
| Humphrey(2019) | Qatari | NM | Eastern Mediterranean | 2013-2016 | General population | simple random | Blood donor center | ELISA-IgG | ≥18 | 100 | 82/1992(4.1) | NM | M |
| Kuan(2016) | Nicaragua | NM | the Americas | 2014-2015 | Age-specific subgroups | simple random | Population | ELISA-IgG | 2-14 | 49.9 | 205/3362(6.1) | 120/205(58.5) | H |
| Kuan(2016) | Nicaragua | NM | the Americas | 2015 | General population | stratified | Population | ELISA-IgG | >15 | 27.6 | 111/848(13.1) | 72/111(64.9) | H |
| Kumar(2011) | India | Rural | South-East Asia Region | 2009 | General population | simple random | Population | IFA-IgG | 14-70 | 37.0 | 260/381(68.2) | 10/260(3.8) | M |
| Kuniholm(2006) | Cameroon | Rural | African | 2002-2003 | General population | simple random | Population | PRNT | >16 | 55.9 | 119/256(46.5) | NM | M |
| Laoprasopwattana(2016) | Thailand | NM | South-East Asia Region | 2009-2010 | Pregnant women | simple random | Hospital | IFA,ELISA-IgG,IgM | >15 | 0 | 227/319(71.2) | 214/227(94.3) | M |
| Lozier(2018) | America | Urban | the Americas | 2016 | General population | convenience | Household | ELISA-IgM,RT-PCR, | all ages | 37.3 | 5/367(0.5) | NM | M |
| Mease(2011) | Kenya | Rural | African | 2004 | General population | multistage | Population | ELISA-IgG | >18 | 35.9 | 383/1141(33.6) | NM | M |
| Moro(2010) | Italy | Urban | European | 2007 | General population | simple random | Population | IFA-IgG | all ages | 47.7 | 33/325(10.2) | 6/33(18) | H |
| Ochieng(2015) | Kenya | Both | African | 2007 | General population | multistage | Household | ELISA-IgG | 15-64 | 39.4 | 10/909(1.1) | NM | M |
| Padbidri(2002) | India | NM | South-East Asia Region | 1988-1989 | General population | simple random | Not specified | HI | all ages | 56.5 | 64/2180(2.9) | NM | M |
| Porter(2004) | Indonesia | NM | South-East Asia Region | 1999 | Others | NM | Not specified | ELISA-IgG,IgM | NM | NM | 77/199(38.7) | NM | M |
| Quan(2018) | Vietnam | NM | Western Pacific | 2015 | Others | NM | Hospital | ELISA-IgG | all ages | NM | 73/546(13.4) | NM | L |
| Rogier(2018) | Haiti | Both | the Americas | 2014-2015 | General population | simple random | Household | Bead-based IgG | 1-99 | NM | 2570/4438(57.9) | NM | H |
| Schwarz(2012) | Madagascar | Urban | African | 2010 | Pregnant women | NM | Center recruitment | IFA-IgG,IgM | 12-50 | 0 | 154/1244(12.4) | 30/144(20.8) | M |
| Seck(2019) | Senegal | Rural | African | 2014 | General population | snowball | Not specified | Bead-based IgG | 1-80 | 43.5 | 39/1434(2.7) | NM | M |
| Sergon(2008) | Kenya | Both | African | 2004 | General population | multistage | Population | ELISA-IgG,IgM | 1-80 | 33.0 | 215/288(74.7) | 100/215(46.5) | H |
| Sergon(2007) | Comoros | NM | African | 2005 | General population | multistage | Population | ELISA-IgG,IgM | 5-80 | 40.6 | 209/331(63.1) | 41/209(19.6) | H |
| Sissoko(2008) | Mayotte | NM | African | 2006 | General population | multistage | Household | ELISA-IgG,IgM | >2 | 43.2 | 440/1154(38.1) | 122/440(27.7) | H |
| Slavov(2018) | Brazil | NM | the Americas | 2015 | Blood Donors | NM | Blood donor center | ELISA-IgG, PCR | NM | NM | 1/442(0.2) | NM | L |
| Vilibic(2015) | Croatian | NM | European | 2011-2012 | Others | simple random | Center recruitment | ELISA-IgG,IgM | 3-88 | NM | 9/1008(0.9) | NM | M |
| Villarroel(2018) | Bolivia | NM | the Americas | 2016-2017 | Blood Donors | NM | Blood donor center | ELISA-IgG | NM | NM | 102/449(22.7) | NM | H |
| Vongpunsawad(2017) | Thailand | NM | South-East Asia Region | 2014 | Others | simple random | Hospital | ELISA-IgG | 0.5-60 | 47.8 | 224/835(26.8) | NM | H |
| Andayi(2014) | Djibouti | NM | Eastern Mediterranean | 2010-2011 | General population | NM | Household | ELISA-IgG | all ages | 45.4 | 24/914(2.6) | NM | M |
| Atalay(2017) | Turkey | NM | European | 2015 | Blood Donors | NM | Blood donor center | ELISA-IgG | NM | 37.4 | 2/500(0.4) | NM | M |
| Gallian(2017) | French Caribbean islands | NM | the Americas | 2015 | Blood Donors | NM | Blood donor center | ELISA-IgG | 18-70 | 46.4 | 782/1754(44.6) | NM | M |
| Gallian(2017) | French Caribbean islands | NM | the Americas | 2014-2015 | Blood Donors | NM | Blood donor center | ELISA-IgG | 18-70 | 46.2 | 1548/8653(17.9) | NM | M |
| LaBeaud(2015) | Kenya | Rural | African | 2009 | General population | cluster | Previous studies | ELISA-IgG | >1 | NM | 486/1848(26.3) | NM | H |
| Rodriguez-Barraquer(2015) | India | NM | South-East Asia Region | 2011 | Age-specific subgroups | cluster | Household | ELISA-IgG | 5-40 | 45.0 | 439/1010(43.5) | 264/439(60.1) | H |
| Simmons(2016) | America | NM | the Americas | 2015 | Blood Donors | NM | Blood donor center | ELISA-IgG | NM | 35.4 | 242/1031(23.5) | NM | M |
| Moss(2018) | Mali | NM | African | NM | School children | stratified | School | MBA-IgG | 4-17 | NM | 50/805(6.2) | NM | M |

Table C: Characteristics of studies included in the systematic review and meta-analysis for ZIKA.

| **First author(Publication Year)** | **Country** | **Rural or Urban** | **WHO Rigion** | **Study Period** | **Population type** | **Sample method** | **Recruitment** | **Laboratory test** | **Age range** | **Male proportion (%)** | **ZIKA** | | **Bias quality** |
| --- | --- | --- | --- | --- | --- | --- | --- | --- | --- | --- | --- | --- | --- |
|  |  |  |  |  |  |  |  |  |  |  | **Prevalence (%)** | **Inapparent proportion (%)** |  |
| Alayed(2018) | Saudi Arabia | Both | Eastern Mediterranean | 2016-2017 | Pregnant women | convenience | Hospital | ELISA-IgG,IgM，RT-PCR | NM | 0 | 52/410 (12.7) | 52/52(100) | M |
| Flamand(2019) | French Guiana | Urban | the Americas | 2017 | General population | simple random | Household | MIA-IgG | 2-75 | 41.1 | 628/2697(23.3) | 468/628(74.5) | M |
| Lozier(2018) | America | Urban | the Americas | 2016 | General population | convenience | Household | ELISA-IgM,RT-PCR, | all ages | 37.3 | 114/367(31.1) | 65/114(57.0) | M |
| Mathé(2018) | Nigeria | NM | African | 2016 | Others | purposive | Hospital | ELISA-IgG,IgM | NM | 16.0 | 48/468(10.2) | 8/48(16.7) | M |
| Pastorino(2019) | Laos | NM | Western Pacific | 2003-2004 | Blood Donors | NM | Blood donor center | ELISA-IgG | 16-63 | 79.8 | 62/359(17.3) | NM | M |
| Pastorino(2019) | Laos | NM | Western Pacific | 2015 | Blood Donors | NM | Blood donor center | ELISA-IgG | 17-79 | 62.4 | 192/687(27.9) | NM | M |
| Sam(2019) | Malaysia | Urban | Western Pacific | 2012,2014,2015,2017 | Others | NM | Hospital | NS1 BOB assay, NA | all ages | 57.8 | 82/1085(7.6) | NM | M |
| Schwarz(2017) | Madagascar | Urban | African | 2010 | Pregnant women | NM | Center recruitment | ELISA-IgG,IgM | NM | 0 | 6/1216(0.5) | NM | M |
| Seruyange(2018) | Rwanda | NM | African | 2015 | Blood Donors | NM | Blood donor center | ELISA-IgG | 17-62 | 65.3 | 12/874(1.4) | NM | H |
| Villarroel(2018) | Bolivia | NM | the Americas | 2016-2017 | Blood Donors | NM | Blood donor center | ELISA-IgG | NM | NM | 213/814(26.2) | NM | H |
| Willcox(2018) | Congo | Both | African | 2013-2014 | Age-specific subgroups | simple random | Household | ELISA-IgG | 0.5-5 | NM | 34/978(3.5) | NM | M |
| Zambrana(2018) | Nicaragua | NM | the Americas | 2017 | Age-specific subgroups | NM | Previous studies | ELISA-IgG | 2-14 | 50.5 | 1346/3740(36.0) | NM | M |
| Zambrana(2018) | Nicaragua | NM | the Americas | 2017 | General population | NM | Previous studies | ELISA-IgG | 15-80 | 28.3 | 601/1074(56.4) | NM | M |
| Babaniyi(2014) | Zambia | Rural | African | NM | General population | multistage | Population | ELISA-IgG | all ages | 46.7 | 217/3625(6.0) | NM | M |

References:

1. Alayed MS, Qureshi MA, Ahmed S, Alqahtani AS, Al-qahtani AM, Alshaybari K, et al. Seroprevalence of Zika virus among asymptomatic pregnant mothers and their newborns in the Najran region of southwest Saudi Arabia. Ann Saudi Med. 2018;38:408-412.

2. Al-Azraqi TA, El Mekki AA, Mahfouzc AA. Seroprevalence of dengue virus infection in Aseer and Jizan regions, Southwestern Saudi Arabia. Trans R Soc Trop Med Hyg. 2013;107:368-371.

3. Al-Raddadi R, Alwafi O, Shabouni O, Akbar N, Alkhalawi M, Ibrahim A, et al. Seroprevalence of dengue fever and the associated sociodemographic, clinical, and environmental factors in Makkah, Madinah, Jeddah, and Jizan, Kingdom of Saudi Arabia. Acta Trop. 2019;189:54-64.

4. Amaya-Larios IY, Rojas-Russell M, López-Cervantes M, Castro-Porras L, Castro-Borbonio MV, Sarti E, et al. Seroprevalence of dengue in school children in Mexico ages 6-17 years, 2016. Trans R Soc Trop Med Hyg. 2018;112:223-229.

5. Ang LW, Cutter J, James L, Goh KT. Seroepidemiology of dengue virus infection in the adult population in tropical Singapore. Epidemiol Infect. 2015;143:1585-1593.

6. Ang LW, Kam YW, Lin C, Krishnan PU, Tay J, Ng LC, et al. Seroprevalence of antibodies against chikungunya virus in Singapore resident adult population. PLoS Negl Trop Dis. 2017;11.

7. Argolo AFLT, Féres VCR, Silveira LA, Oliveira ACM, Pereira LA, Júnior JBS, et al. Prevalence and incidence of dengue virus and antibody placental transfer during late pregnancy in central Brazil. BMC Infect Dis. 2013;13.

8. Ashshi AM. The prevalence of dengue virus serotypes in asymptomatic blood donors reveals the emergence of serotype 4 in Saudi Arabia. Virol J. 2017;14.

9. Auerswald H, De Jesus A, Seixas G, Nazareth T, In S, Mao S, et al. First dengue virus seroprevalence study on Madeira Island after the 2012 outbreak indicates unreported dengue circulation. Parasites and Vectors. 2019;12.

10. Azami NAM, Salleh SA, Shah SA, Neoh HM, Othman Z, Zakaria SZS, et al. Emergence of chikungunya seropositivity in healthy Malaysian adults residing in outbreak-free locations: Chikungunya seroprevalence results from the Malaysian Cohort. BMC Infect Dis. 2013;13.

11. Bacci A, Marchi S, Fievet N, Massougbodji A, Perrin RX, Chippaux JP, et al. High seroprevalence of chikungunya virus antibodies among pregnant women living in an urban area in Benin, West Africa. American Journal of Tropical Medicine and Hygiene. 2015;92:1133-1136.

12. Barakat AM, Smura T, Kuivanen S, Huhtamo E, Kurkela S, Putkuri N, et al. The presence and seroprevalence of arthropod-borne viruses in Nasiriyah Governorate, Southern Iraq: A cross-sectional study. American Journal of Tropical Medicine and Hygiene. 2016;94:794-799.

13. Braga C, Luna CF, Martelli CMT, Souza WVD, Cordeiro MT, Alexander N, et al. Seroprevalence and risk factors for dengue infection in socio-economically distinct areas of Recife, Brazil. Acta Trop. 2010;113:234-240.

14. Brunkard JM, Robles Lopez JL, Ramirez J, Cifuentes E, Rothenberg SJ, Hunsperger EA, et al. Dengue fever seroprevalence and risk factors, Texas-Mexico border, 2004. Emerging infectious diseases. 2007;13:1477-1483.

15. Campbell CA, George A, Salas RA, Williams SA, Doon R, Chadee DD. Seroprevalence of dengue in Trinidad using rapid test kits: A cord blood survey. Acta Trop. 2007;101:153-158.

16. Carabali M, Lim JK, Velez DC, Trujillo A, Egurrola J, Lee KS, et al. Dengue virus serological prevalence and seroconversion rates in children and adults in Medellin, Colombia: implications for vaccine introduction. International Journal of Infectious Diseases. 2017;58:27-36.

17. Chiaravalloti-Neto F, da Silva RA, Zini N, da Silva GCD, da Silva NS, Parra MCP, et al. Seroprevalence for dengue virus in a hyperendemic area and associated socioeconomic and demographic factors using a cross-sectional design and a geostatistical approach, state of SAo Paulo, Brazil. BMC Infect Dis. 2019;19.

18. Chien YW, Huang HM, Ho TC, Tseng FC, Ko NY, Ko WC, et al. Seroepidemiology of dengue virus infection among adults during the ending phase of a severe dengue epidemic in southern Taiwan, 2015. BMC Infect Dis. 2019;19.

19. Chow VTK, Lim CS, Phoon MC, Tan HC. A seroprevalence survey of dengue virus infection in healthy Singapore university undergraduates by enzyme immunoassay and plaque reduction neutralization test. Dengue Bulletin. 2005;29:97-105.

20. Cortes-Escamilla A, López-Gatell H, Sánchez-Alemán MÁ, Hegewisch-Taylor J, Hernández-Ávila M, Alpuche-Aranda CM. The hidden burden of Chikungunya in central Mexico: results of a small-scale serosurvey. Salud Publica Mex. 2018;60:63-70.

21. Cunha RV, Trinta KS, Montalbano CA, Sucupira MVF, de Lima MM, Marques E, et al. Seroprevalence of Chikungunya Virus in a Rural Community in Brazil. PLoS Negl Trop Dis. 2017;11.

22. Darcy A, Clothier H, Phillips D, Bakote'e B, Stewart T. Solomon Islands dengue seroprevalence study--previous circulation of dengue confirmed. Papua and New Guinea medical journal. 2001;44:43-47.

23. de Almeida D, Dantas DD, Resque RL, Souza KHD, Yoshida MT. Profile of blood donors and seroepidemiology of dengue in a blood center in the Brazilian Amazon. Mundo Da Saude. 2018;42:893-904.

24. Demanou M, Antonio-Nkondjio C, Ngapana E, Rousset D, Paupy C, Manuguerra JC, et al. Chikungunya outbreak in a rural area of Western Cameroon in 2006: A retrospective serological and entomological survey. BMC research notes. 2010;3:128.

25. Dhanoa A, Hassan SS, Jahan NK, Reidpath DD, Fatt QK, Ahmad MP, et al. Seroprevalence of dengue among healthy adults in a rural community in Southern Malaysia: A pilot study. Infectious Diseases of Poverty. 2018;7.

26. Dhar-Chowdhury P, Paul KK, Haque CE, Hossain S, Lindsay LR, Dibernardo A, et al. Dengue seroprevalence, seroconversion and risk factors in Dhaka, Bangladesh. PLoS Negl Trop Dis. 2017;11.

27. Dias JP, Costa MDN, Campos GS, Paixao ES, Natividade MS, Barreto FR, et al. Seroprevalence of Chikungunya Virus in 2 Urban Areas of Brazil 1 Year after Emergence. Emerg Infect Dis. 2018;24:617-624.

28. Eldigail MH, Adam GK, Babiker RA, Khalid F, Adam IA, Omer OH, et al. Prevalence of dengue fever virus antibodies and associated risk factors among residents of El-Gadarif state, Sudan. BMC public health. 2018;18:921.

29. Ellis EM, Neatherlin JC, Delorey M, Ochieng M, Mohamed AH, Mogeni DO, et al. A Household Serosurvey to Estimate the Magnitude of a Dengue Outbreak in Mombasa, Kenya, 2013. PLoS Negl Trop Dis. 2015;9.

30. Flamand C, Bailly S, Fritzell C, Berthelot L, Vanhomwegen J, Salje H, et al. Impact of Zika virus emergence in French Guiana: A large general population seroprevalence survey. The Journal of infectious diseases. 2019.

31. Fox-Lewis A, Hopkins J, Sar P, Sao S, Pheaktra N, Day NPJ, et al. Seroprevalence of dengue virus and rickettsial infections in cambodian children. American Journal of Tropical Medicine and Hygiene. 2019;100:635-638.

32. Gabor JJ, Schwarz NG, Esen M, Kremsner PG, Grobusch MP. Dengue and chikungunya seroprevalence in Gabonese infants prior to major outbreaks in 2007 and 2010: A sero-epidemiological study. Travel Med Infect Dis. 2016;14:26-31.

33. Garg S, Chakravarti A, Singh R, Masthi NRR, Goyal RC, Jammy GR, et al. Dengue serotype-specific seroprevalence among 5-to 10-year-old children in India: a community-based cross-sectional study. International Journal of Infectious Diseases. 2017;54:25-30.

34. Gay N, Rousset D, Huc P, Matheus S, Ledrans M, Rosine J, et al. Seroprevalence of asian lineage chikungunya virus infection on saint martin Island, 7 months after the 2013 emergence. American Journal of Tropical Medicine and Hygiene. 2016;94:393-396.

35. Gerardin P, Guernier V, Perrau J, Fianu A, Le Roux K, Grivard P, et al. Estimating Chikungunya prevalence in La Reunion Island outbreak by serosurveys: Two methods for two critical times of the epidemic. BMC Infect Dis. 2008;8.

36. Guo RN, Lin JY, Li LH, Ke CW, He JF, Zhong HJ, et al. The prevalence and endemic nature of dengue infections in Guangdong, South China: An epidemiological, serological, and etiological study from 2005-2011. PloS one. 2014;9.

37. Hennessey MJ, Ellis EM, Delorey MJ, Panella AJ, Kosoy OI, Kirking HL, et al. Seroprevalence and Symptomatic Attack Rate of Chikungunya Virus Infection, United States Virgin Islands, 2014-2015. The American journal of tropical medicine and hygiene. 2018;99:1321-1326.

38. Honório NA, Nogueira RMR, Codeço CT, Carvalho MS, Cruz OG, Magalhães MDAFM, et al. Spatial evaluation and modeling of dengue seroprevalence and vector density in Rio de Janeiro, Brazil. PLoS Negl Trop Dis. 2009;3.

39. Humphrey JM, Al-Absi ES, Hamdan MM, Okasha SS, Al-Trmanini DM, El-Dous HG, et al. Dengue and chikungunya seroprevalence among Qatari nationals and immigrants residing in Qatar. PloS one. 2019;14.

40. Iturrino-Monge R, Avila-Agüero ML, Avila-Agüero CR, Moya-Moya T, Cañas-Coto A, Camacho-Badilla K, et al. Seroprevalence of dengue virus antibodies in asymptomatic Costa Rican children, 2002-2003: A pilot study. Revista Panamericana de Salud Publica/Pan American Journal of Public Health. 2006;20:39-43.

41. Jamjoom GA, Azhar EI, Kao MA, Radadi RM. Seroepidemiology of asymptomatic dengue virus infection in Jeddah, Saudi Arabia. Virology: Research and Treatment. 2016;2016:1-7.

42. Jeewandara C, Gomes L, Paranavitane SA, Tantirimudalige M, Panapitiya SS, Jayewardene A, et al. Change in dengue and Japanese encephalitis seroprevalence rates in Sri Lanka. PloS one. 2015;10.

43. Jing Q, Li Y, Liu J, Jiang L, Chen Z, Su W, et al. Dengue Underestimation in Guangzhou, China: Evidence of Seroprevalence in Communities With No Reported Cases Before a Large Outbreak in 2014. Open forum infectious diseases. 2019;6:ofz256.

44. Kuan G, Ramirez S, Gresh L, Ojeda S, Melendez M, Sanchez N, et al. Seroprevalence of Anti-Chikungunya Virus Antibodies in Children and Adults in Managua, Nicaragua, After the First Chikungunya Epidemic, 2014-2015. PLoS Negl Trop Dis. 2016;10.

45. Kumar NP, Suresh A, Vanamail P, Sabesan S, Krishnamoorthy KG, Mathew J, et al. Chikungunya virus outbreak in Kerala, India, 2007: A seroprevalence study. Mem Inst Oswaldo Cruz. 2011;106:912-916.

46. Kuniholm MH, Wolfe ND, Huang CYH, Mpoudi-Ngole E, Tamoufe U, Burke DS, et al. Seroprevalence and distribution of Flaviviridae, Togaviridae, and Bunyaviridae arboviral infections in rural Cameroonian adults. American Journal of Tropical Medicine and Hygiene. 2006;74:1078-1083.

47. Laoprasopwattana K, Suntharasaj T, Petmanee P, Suddeaugrai O, Geater A. Chikungunya and dengue virus infections during pregnancy: Seroprevalence, seroincidence and maternal-fetal transmission, southern Thailand, 2009-2010. Epidemiol Infect. 2016;144:381-388.

48. L'Azou M, Jean-Marie J, Bessaud M, Cabié A, Cesaire R, De Lamballerie X, et al. Dengue seroprevalence in the French West Indies: A prospective study in adult blood donors. American Journal of Tropical Medicine and Hygiene. 2015;92:1137-1140.

49. Leder K, Mutsch M, Schlagenhauf P, Luxemburger C, Torresi J. Seroepidemiology of dengue in travellers: A paired sera analysis. Travel Med Infect Dis. 2013;11:210-213.

50. Lee P, Yeung ACM, Chen Z, Chan MCW, Sze KH, Chan PKS. Age-specific seroprevalence of dengue infection in Hong Kong. J Med Virol. 2018;90:1427-1430.

51. Leslie T, Martin NJ, Jack-Roosberg C, Odongo G, Beausoleil E, Tuck J, et al. Dengue serosurvey in Sint Eustatius. PloS one. 2014;9.

52. Liu JD, Deng Y, Jing QL, Chen XS, Du ZC, Liang TZ, et al. Dengue Infection Spectrum in Guangzhou: A Cross-Sectional Seroepidemiology Study among Community Residents between 2013 and 2015. International Journal of Environmental Research and Public Health. 2018;15.

53. Liu L, Huang J, Zhong M, Yuan K, Chen Y. Seroprevalence of Dengue Virus Among Pregnant Women in Guangdong, China. Viral Immunol. 2019.

54. Lo CLH, Yip SP, Leung PHM. Seroprevalence of dengue in the general population of Hong Kong. Trop Med Int Health. 2013;18:1097-1102.

55. Low SL, Lam S, Wong WY, Teo D, Ng LC, Tan LK. Dengue seroprevalence of healthy adults in Singapore: Serosurvey among blood donors, 2009. American Journal of Tropical Medicine and Hygiene. 2015;93:40-45.

56. Lozier MJ, Burke RM, Lopez J, Acevedo V, Amador M, Read JS, et al. Differences in prevalence of symptomatic zika virus infection, by age and sex—puerto rico, 2016. Journal of Infectious Diseases. 2018;217:1678-1689.

57. Mai VQ, Mai TTX, Tam NLM, Nghia LT, Komada K, Murakami H. Prevalence and Risk Factors of Dengue Infection in Khanh Hoa Province, Viet Nam: A Stratified Cluster Sampling Survey. J Epidemiol. 2018;28:488-497.

58. Malavige GN, Fernando S, Aaskov J, Sivayogan S, Dissanayaka T, Peelawattage MK, et al. Seroprevalence of anti-dengue virus antibodies in children in Colombo district, Sri Lanka. Dengue Bulletin. 2006;30:68-71.

59. Martins AC, Pereira TM, Oliart-Guzmán H, Delfino BM, Mantovani SAS, Braña AM, et al. Seroprevalence and seroconversion of dengue and implications for clinical diagnosis in amazonian children. Interdiscip Perspect Infect Dis. 2014;2014.

60. Mathé P, Egah DZ, Müller JA, Shehu NY, Obishakin ET, Shwe DD, et al. Low Zika virus seroprevalence among pregnant women in North Central Nigeria, 2016. Journal of Clinical Virology. 2018;105:35-40.

61. Mazaba-Liwewe ML, Siziya S, Monze M, Mweene-Ndumba I, Masaninga F, Songolo P, et al. First sero-prevalence of dengue fever specific immunoglobulin G antibodies in Western and North-Western provinces of Zambia: A population based cross sectional study. Virol J. 2014:135.

62. Mease LE, Coldren RL, Musila LA, Prosser T, Ogolla F, Ofula VO, et al. Seroprevalence and distribution of arboviral infections among rural Kenyan adults: A cross-sectional study. Virol J. 2011;8.

63. Messenger AM, Barr KL, Weppelmann TA, Barnes AN, Anderson BD, Okech BA, et al. Serological evidence of ongoing transmission of dengue virus in permanent residents of Key West, Florida. Vector-Borne and Zoonotic Diseases. 2014;14:783-787.

64. Mishra AC, Arankalle VA, Gadhave SA, Mahadik PH, Shrivastava S, Bhutkar M, et al. Stratified sero-prevalence revealed overall high disease burden of dengue but suboptimal immunity in younger age groups in Pune, India. PLoS Negl Trop Dis. 2018;12.

65. Mohammed H, Tomashek KM, Stramer SL, Hunsperger E. Prevalence of anti-dengue immunoglobulin G antibodies among American Red Cross blood donors in Puerto Rico, 2006. Transfusion. 2012;52:1652-1656.

66. Mohsin SN, Ghafoor F, Saleem M, Ghous R, Aasim M. Seroprevalence of asymptomatic dengue infection in children in Lahore. Epidemiol Infect. 2016;144:2276-2282.

67. Moro ML, Gagliotti C, Silvi G, Angelini R, Sambri V, Rezza G, et al. Chikungunya Virus in North-Eastern Italy: A Seroprevalence Survey. American Journal of Tropical Medicine and Hygiene. 2010;82:508-511.

68. Murhekar MV, Kamaraj P, Kumar MS, Khan SA, Allam RR, Barde P, et al. Burden of dengue infection in India, 2017: a cross-sectional population based serosurvey. The Lancet Global Health. 2019;7:e1065-e1073.

69. Nujum ZT, Saritha N, Prathibha Raj MR, Gayathri AV, Nirmala C, Vijayakumar K, et al. Seroprevalence of dengue infection in pregnant women and placental antibody transfer. Medical Journal Armed Forces India. 2019;75:90-95.

70. Obaidat MM, Roess AA. First report on seroprevalence and risk factors of dengue virus in Jordan. Trans R Soc Trop Med Hyg. 2018;112:279-284.

71. Ochieng C, Ahenda P, Vittor AY, Nyoka R, Gikunju S, Wachira C, et al. Seroprevalence of infections with dengue, Rift Valley fever and chikungunya viruses in Kenya, 2007. PloS one. 2015;10.

72. Overbosch FW, van den Hoek A, Schinkel J, Sonder GJB. High prevalence of previous dengue virus infection among first-generation Surinamese immigrants in the Netherlands. BMC Infect Dis. 2014;14.

73. Pacsa AS, Chaturvedi UC, Mustafa AS. Seroprevalence of three emerging arboviral infections in Kuwaiti nationals. East Mediterr Health J. 2003;9:266-273.

74. Padbidri VS, Wairagkar NS, Joshi GD, Umarani UB, Risbud AR, Gaikwad DL, et al. A serological survey of arboviral diseases among the human population of the Andaman and Nicobar Islands, India. The Southeast Asian journal of tropical medicine and public health. 2002;33:794-800.

75. Pastorino B, Sengvilaipaseuth O, Chanthongthip A, Vongsouvath M, Souksakhone C, Mayxay M, et al. Low Zika Virus Seroprevalence in Vientiane, Laos, 2003-2015. American Journal of Tropical Medicine and Hygiene. 2019;100:639-642.

76. Pavía-Ruz N, Barrera-Fuentes GA, Villanueva-Jorge S, Che-Mendoza A, Campuzano-Rincón JC, Manrique-Saide P, et al. Dengue seroprevalence in a cohort of schoolchildren and their siblings in Yucatan, Mexico (2015-2016). PLoS Negl Trop Dis. 2018;12.

77. Pavia-Ruz N, Rojas DP, Villanueva S, Granja P, Balann-May A, Longini IM, et al. Seroprevalence of Dengue Antibodies in Three Urban Settings in Yucatan, Mexico. American Journal of Tropical Medicine and Hygiene. 2018;98:1202-1208.

78. Pem-Novosel I, Vilibic-Cavlek T, Gjenero-Margan I, Kaic B, Babic-Erceg A, Merdic E, et al. Dengue virus infection in Croatia: seroprevalence and entomological study. The new microbiologica. 2015;38:97-100.

79. Pereira A, de Siqueira TR, Prado AAD, da Silva CAV, Moraes TDS, Aleixo AA, et al. High prevalence of dengue antibodies and the arginine variant of the Fc gamma RIIa polymorphism in asymptomatic individuals in a population of Minas Gerais State, Southeast Brazil. Immunogenetics. 2018;70:355-362.

80. Piedrahita LD, Salas IYA, Marin K, Trujillo AI, Osorio JE, Arboleda-Sanchez SO, et al. Risk Factors Associated with Dengue Transmission and Spatial Distribution of High Seroprevalence in Schoolchildren from the Urban Area of Medellin, Colombia. Can J Infect Dis Med Microbiol. 2018.

81. Porter KR, Tan R, Istary Y, Suharyono W, Sutaryo, Widjaja S, et al. A serological study of Chikungunya virus transmission in Yogyakarta, Indonesia: Evidence for the first outbreak since 1982. Southeast Asian Journal of Tropical Medicine and Public Health. 2004;35:408-415.

82. Prayitno A, Taurel AF, Nealon J, Satari HI, Karyanti MR, Sekartini R, et al. Dengue seroprevalence and force of primary infection in a representative population of urban dwelling Indonesian children. PLoS Negl Trop Dis. 2017;11.

83. Quan TM, Phuong HT, Vy NHT, Thanh NTL, Lien NTN, Hong TTK, et al. Evidence of previous but not current transmission of chikungunya virus in southern and central Vietnam: Results from a systematic review and a seroprevalence study in four locations. PLoS Negl Trop Dis. 2018;12.

84. Quyen DL, Thanh Le N, Van Anh CT, Nguyen NB, Hoang DV, Montgomery JL, et al. Epidemiological, Serological, and Virological Features of Dengue in Nha Trang City, Vietnam. The American journal of tropical medicine and hygiene. 2018;98:402-409.

85. Ramos MM, Mohammed H, Zielinski-Gutierrez E, Hayden MH, Lopez JLR, Fournier M, et al. Epidemic dengue and dengue hemorrhagic fever at the Texas-Mexico border: Results of a household-based seroepidemiologic survey, December 2005. American Journal of Tropical Medicine and Hygiene. 2008;78:364-369.

86. Ranjan P, Natarajan V, Bajpai M, Gupta E. High seroprevalence of dengue virus infection in blood donors from Delhi: A single centre study. Journal of Clinical and Diagnostic Research. 2016;10:DC08-DC10.

87. Ratnam I, Black J, Leder K, Biggs BA, Matchett E, Padiglione A, et al. Incidence and seroprevalence of dengue virus infections in Australian travellers to Asia. European Journal of Clinical Microbiology and Infectious Diseases. 2012;31:1203-1210.

88. Rioth M, Beauharnais CA, Noel F, Ikizler MR, Mehta S, Zhu YW, et al. Serologic Imprint of Dengue Virus in Urban Haiti: Characterization of Humoral Immunity to Dengue in Infants and Young Children. American Journal of Tropical Medicine and Hygiene. 2011;84:630-636.

89. Rogier EW, Moss DM, Mace KE, Chang M, Jean SE, Bullard SM, et al. Use of bead-based serologic assay to evaluate chikungunya virus epidemic, Haiti. Emerg Infect Dis. 2018;24:995-1001.

90. Sacramento RHM, de Carvalho Araújo FM, Lima DM, Alencar CCH, Martins VEP, Araújo LV, et al. Dengue Fever and Aedes aegypti in indigenous Brazilians: seroprevalence, risk factors, knowledge and practices. Tropical Medicine and International Health. 2018;23:596-604.

91. Sam IC, Montoya M, Chua CL, Chan YF, Pastor A, Harris E. Low seroprevalence rates of Zika virus in Kuala Lumpur, Malaysia. Trans R Soc Trop Med Hyg. 2019.

92. Sanchez-Vegas C, Hamer DH, Chen LH, Wilson ME, Benoit C, Hunsperger E, et al. Prevalence of dengue virus infection in US travelers who have lived in or traveled to dengue-endemic countries. J Travel Med. 2013;20:352-360.

93. Schwarz NG, Girmann M, Randriamampionona N, Bialonski A, Maus D, Krefis AC, et al. Seroprevalence of antibodies against chikungunya, dengue, and Rift Valley fever viruses after febrile illness outbreak, Madagascar. Emerg Infect Dis. 2012;18:1780-1786.

94. Schwarz NG, Mertens E, Winter D, Maiga-Ascofare O, Dekker D, Jansen S, et al. No serological evidence for Zika virus infection and low specificity for anti-Zika virus ELISA in malaria positive individuals among pregnant women from Madagascar in 2010. PloS one. 2017;12.

95. Seck MC, Badiane AS, Thwing J, Moss D, Fall FB, Gomis JF, et al. Serological data shows low levels of chikungunya exposure in senegalese nomadic pastoralists. Pathogens. 2019;8.

96. Sergon K, Njuguna C, Kalani R, Ofula V, Onyango C, Konongoi LS, et al. Seroprevalence of Chikungunya virus (CHIKV) infection on Lamu Island, Kenya, October 2004. American Journal of Tropical Medicine and Hygiene. 2008;78:333-337.

97. Sergon K, Yahaya AA, Brown J, Bedja SA, Mlindasse M, Agata N, et al. Seroprevalence of Chikungunya virus infection on Grande Comore Island, Union of the Comoros, 2005. American Journal of Tropical Medicine and Hygiene. 2007;76:1189-1193.

98. Seruyange E, Gahutu JB, Muvunyi CM, Katare S, Ndahindwa V, Sibomana H, et al. Seroprevalence of Zika virus and Rubella virus IgG among blood donors in Rwanda and in Sweden. J Med Virol. 2018;90:1290-1296.

99. Shah PS, Alagarasu K, Karad S, Deoshatwar A, Jadhav SM, Raut T, et al. Seroprevalence and incidence of primary dengue infections among children in a rural region of Maharashtra, Western India. BMC Infect Dis. 2019;19.

100. Shah PS, Deoshatwar A, Karad S, Mhaske S, Singh A, Bachal RV, et al. Seroprevalence of dengue in a rural and an urbanized village: A pilot study from rural western India. J Vector Borne Dis. 2017;54:172-176.

101. Shaukat S, Ul Jawad SMT, Kashif M. PREVALENCE OF DENGUE FEVER IN THE POPULATION OF MULTAN. Indo Am J Pharm Sci. 2018;5:7088-7091.

102. Sissoko D, Ezzedine K, Giry C, Moendandzé A, Lernout T, D'Ortenzio E, et al. Seroepidemiology of Dengue virus in Mayotte, Indian Ocean, 2006. PloS one. 2010;5.

103. Sissoko D, Moendandze A, Malvy D, Giry C, Ezzedine K, Solet JL, et al. Seroprevalence and risk factors of Chikungunya virus infection in Mayotte, Indian Ocean, 2005-2006: A population- based survey. PloS one. 2008;3.

104. Slavov SN, Cilião-Alves DC, Gonzaga FAC, Moura DR, de Moura ACAM, de Noronha LAG, et al. Dengue seroprevalence among asymptomatic blood donors during an epidemic outbreak in Central-West Brazil. PloS one. 2019;14.

105. Slavov SN, Otaguiri KK, Bianquini ML, Bitencourt HTO, Chagas MCM, Guerreiro DSS, et al. Seroprevalence of Chikungunya virus in blood donors from Northern and Southeastern Brazil. Hematology, transfusion and cell therapy. 2018;40:358-362.

106. Soghaier MA, Himatt S, Osman KE, Okoued SI, Seidahmed OE, Beatty ME, et al. Cross-sectional community-based study of the socio-demographic factors associated with the prevalence of dengue in the eastern part of Sudan in 2011. BMC public health. 2015;15:558.

107. Soghaier MA, Mahmood SF, Pasha O, Azam SI, Karsani MM, Elmangory MM, et al. Factors associated with dengue fever IgG sero-prevalence in South Kordofan State, Sudan, in 2012: Reporting prevalence ratios. Journal of infection and public health. 2014;7:54-61.

108. Succo T, Noel H, Nikolay B, Maquart M, Cochet A, Leparc-Goffart I, et al. Dengue serosurvey after a 2-month long outbreak in Nimes, France, 2015: was there more than met the eye? Eurosurveillance. 2018;23:25-34.

109. Tan PC, Rajasingam G, Devi S, Omar SZ. Dengue infection in pregnancy - Prevalence, vertical transmission, and pregnancy outcome. Obstet Gynecol. 2008;111:1111-1117.

110. Thisyakorn U, Srettakraikul K, Hemungkorn M, Thisyakorn C. Seroepidemiology of dengue virus infection in HIV-infected children in comparison to healthy children. Journal of the Medical Association of Thailand. 2016;99:175-181.

111. Tiong V, Abd-Jamil J, Zan HAM, Abu-Bakar RS, Ew CL, Jafar FL, et al. Evaluation of land cover and prevalence of dengue in Malaysia. Tropical Biomedicine. 2015;32:587-597.

112. Tissera HA, De Silva AD, Abeysinghe MRN, de Silva AM, Palihawadana P, Gunasena S, et al. Dengue Surveillance in Colombo, Sri Lanka: Baseline seroprevalence among children. Procedia in Vaccinology. 2010;2:107-110.

113. Tsai JJ, Liu CK, Tsai WY, Liu LT, Tyson J, Tsai CY, et al. Seroprevalence of dengue virus in two districts of Kaohsiung City after the largest dengue outbreak in Taiwan since World War II. PLoS Negl Trop Dis. 2018;12.

114. Velasco-Salas ZI, Sierra GM, Guzmán DM, Zambrano J, Vivas D, Comach G, et al. Dengue seroprevalence and risk factors for past and recent viral transmission in Venezuela: A comprehensive community-based study. American Journal of Tropical Medicine and Hygiene. 2014;91:1039-1048.

115. Vilibic-Cavlek T, Pem-Novosel I, Kaic B, Babić-Erceg A, Kucinar J, Klobucar A, et al. Seroprevalence and entomological study on chikungunya virus at the Croatian littoral. Acta Microbiologica et Immunologica Hungarica. 2015;62:199-206.

116. Villarroel PMS, Nurtop E, Pastorino B, Roca Y, Drexler JF, Gallian P, et al. Zika virus epidemiology in Bolivia: A seroprevalence study in volunteer blood donors. PLoS Negl Trop Dis. 2018;12.

117. Vongpunsawad S, Intharasongkroh D, Thongmee T, Poovorawan Y. Seroprevalence of antibodies to dengue and chikungunya viruses in Thailand. PloS one. 2017;12.

118. Wilder-Smith A, Foo W, Earnest A, Sremulanathan S, Paton NI. Seroepidemiology of dengue in the adult population of Singapore. Tropical Medicine and International Health. 2004;9:305-308.

119. Wilder-Smith A, Yoksan S, Earnest A, Subramaniam R, Paton NI. Serological evidence for the co-circulation of multiple dengue virus serotypes in Singapore. Epidemiol Infect. 2005;133:667-671.

120. Willcox AC, Collins MH, Jadi R, Keeler C, Parr JB, Mumba D, et al. Seroepidemiology of dengue, zika, and yellow fever viruses among children in the democratic republic of the Congo. American Journal of Tropical Medicine and Hygiene. 2018;99:756-763.

121. Yamashiro T, Disla M, Petit A, Taveras D, Castro-Bello M, Lora-Orste M, et al. Seroprevalence of IgG specific for dengue virus among adults and children in Santo Domingo, Dominican Republic. American Journal of Tropical Medicine and Hygiene. 2004;71:138-143.

122. Yik WY, Ye T, Li WA, Lee CN, Yap G, James L, et al. Seroepidemiology of dengue virus infection among adults in Singapore. Annals of the Academy of Medicine Singapore. 2009;38:667-675.

123. Zambrana JV, Carrillo FB, Burger-Calderon R, Collado D, Sanchez N, Ojeda S, et al. Seroprevalence, risk factor, and spatial analyses of Zika virus infection after the 2016 epidemic in Managua, Nicaragua. Proc Natl Acad Sci U S A. 2018;115:9294-9299.

124. Andayi F, Charrel RN, Kieffer A, Richet H, Pastorino B, Leparc-Goffart I, et al. A sero-epidemiological study of arboviral fevers in Djibouti, Horn of Africa. PLoS neglected tropical diseases. 2014;8:e3299.

125. Atalay T, Kaygusuz S, Azkur AK. A study of the chikungunya virus in humans in Turkey. Turkish journal of medical sciences. 2017;47:1161-1164.

126. Babaniyi O, Mwaba P, Songolo P, Mazaba-Liwewe M, Mweene-Ndumba I, Masaninga F, et al. Seroprevalence of Zika virus infection specific IgG in Western and North-Western Provinces of Zambia. International Journal of Public Health. 2015;4:2326-7291.

127. Gallian P, Leparc-Goffart I, Richard P, Maire F, Flusin O, Djoudi R, et al. Epidemiology of Chikungunya Virus Outbreaks in Guadeloupe and Martinique, 2014: An Observational Study in Volunteer Blood Donors. PLoS neglected tropical diseases. 2017;11:e0005254.

128. LaBeaud AD, Banda T, Brichard J, Muchiri EM, Mungai PL, Mutuku FM, et al. High rates of o'nyong nyong and Chikungunya virus transmission in coastal Kenya. PLoS neglected tropical diseases. 2015;9:e0003436.

129. Rodriguez-Barraquer I, Solomon SS, Kuganantham P, Srikrishnan AK, Vasudevan CK, Iqbal SH, et al. The Hidden Burden of Dengue and Chikungunya in Chennai, India. PLoS neglected tropical diseases. 2015;9:e0003906.

130. Seidahmed OM, Hassan SA, Soghaier MA, Siam HA, Ahmed FT, Elkarsany MM, et al. Spatial and temporal patterns of dengue transmission along a Red Sea coastline: a longitudinal entomological and serological survey in Port Sudan city. PLoS neglected tropical diseases. 2012;6:e1821.

131. Simmons G, Bres V, Lu K, Liss NM, Brambilla DJ, Ryff KR, et al. High Incidence of Chikungunya Virus and Frequency of Viremic Blood Donations during Epidemic, Puerto Rico, USA, 2014. Emerging infectious diseases. 2016;22:1221-1228.

132. Teixeira Mda G, Barreto ML, Costa Mda C, Ferreira LD, Vasconcelos PF, Cairncross S. Dynamics of dengue virus circulation: a silent epidemic in a complex urban area. Tropical medicine & international health : TM & IH. 2002;7:757-762.

133. MossDelynn M, WhitneyMatthew T, ChardAnna N, Victoria T, Seydou D, GoodmanChristin H, et al. Serological Evidence of Dengue and Chikungunya Exposures in Malian Children by Multiplex Bead Assay. International Journal of Tropical Diseases. 2018;1: 1:007.
