# Supplementary material for: The worldwide seroprevalence of DENV, CHIKV and ZIKV infection: A systematic review and meta-analysis
Source: PLoS Negl Trop Dis. 2021 Apr 28;15(4):e0009337. doi: 10.1371/journal.pntd.0009337 (PMC8109817; doi:10.1371/journal.pntd.0009337)
Supplement: S2 Text — Table A. Seroprevalence of DENV infection for developing and developed countries. Table B. Seroprevalence of DENV infection for urban and rural areas. Table C. Time trend of DENV seroprevalence. Table D: Seroprevalence of DENV infection in general population. Table E. Seroprevalence of DENV infection stratified by diagnostic tests. Table F. Seroprevalence of DENV infection stratified by age. Table G. Proportion of DENV inapparent infection. (DOCX) [file pntd.0009337.s002.docx]

S2 Appendix

Table A: Seroprevalence of DENV infection for developing and developed countries .

Table B: Seroprevalence of DENV infection for urban and rural areas.

Table C: Time trend of DENV seroprevalence.

Table D: Seroprevalence of DENV infection in general population .

Table E: Seroprevalence of DENV infection stratified by diagnostic tests .

Table F: Seroprevalence of DENV infection stratified by age.

Table G: Proportion of DENV inapparent infection.

**Table A: Seroprevalence of DENV infection for developing and developed countries.**

| **Regions** | **Developed** | | | | **Developing** | | | |
| --- | --- | --- | --- | --- | --- | --- | --- | --- |
|  | **Number of reports** | **cases** | **samples** | **seroprevalence(%,95%CI)** | **Number of reports** | **cases** | **samples** | **seroprevalence(%,95%CI)** |
| **African** | / | / | / | / | 9 | 1046 | 10801 | 12(8-15) |
| **Eastern Mediterranean** | / | / | / | / | 15 | 5095 | 17912 | 27(21-34) |
| **European** | 3 | 56 | 2100 | 4(0-7) | / | / | / | / |
| **South-East Asia** | / | / | / | / | 17 | 15329 | 29882 | 56(39-73) |
| **the Americas** | 7 | 3504 | 6312 | 42(12-71) | 23 | 11172 | 19245 | 52(35-68) |
| **Western Pacific** | / | / | / | / | 21 | 8964 | 33960 | 27(19-35) |

**Table B: Seroprevalence of DENV infection for urban and rural areas.**

| **Regions** | **Urban** | | | | **Rural** | | | |
| --- | --- | --- | --- | --- | --- | --- | --- | --- |
|  | **Number of reports** | **cases** | **samples** | **seroprevalence(%,95%CI)** | **Number of reports** | **cases** | **samples** | **seroprevalence(%,95%CI)** |
| **African** | 2 | 307 | 2744 | 11(5-17) | 2 | 194 | 1397 | 14(12-16) |
| **Eastern Mediterranean** | 6 | 1919 | 4960 | 34(30-38) | 2 | 43 | 213 | 20(15-26) |
| **European** | / | / | / | / | / | / | / | / |
| **South-East Asia** | 7 | 9959 | 14262 | 65（55-74) | 4 | 3073 | 7467 | 33(21-45) |
| **the Americas** | 13 | 10336 | 16754 | 50(29-70) | 1 | 62 | 280 | 22(17-27） |
| **Western Pacific** | 10 | 3079 | 17927 | 17(9-24) | 3 | 391 | 1915 | 20(7-33） |

**Table C: Time trend of DENV seroprevalence.**

| **Regions** | **2000-2009** | | | | **2010-2019** | | | |
| --- | --- | --- | --- | --- | --- | --- | --- | --- |
|  | **Number of reports** | **cases** | **samples** | **seroprevalence(%,95%CI)** | **Number of reports** | **cases** | **samples** | **seroprevalence(%,95%CI)** |
| **African** | 5 | 619 | 3804 | 15(11-19) | 4 | 493 | 7346 | 7(4-11) |
| **Eastern Mediterranean** | 2 | 111 | 1290 | 10(1-18) | 9 | 3583 | 13135 | 28(22-33) |
| **European** | / | / | / | / | 3 | 56 | 2100 | 4(0-7) |
| **South-East Asia** | 2 | 487 | 3198 | 27(20-75) | 11 | 14375 | 25809 | 64(51-76) |
| **the Americas** | 12 | 5742 | 7471 | 65(51-79) | 17 | 10082 | 19441 | 42(25-59) |
| **Western Pacific** | 7 | 3055 | 9775 | 26(9-42) | 11 | 3839 | 14808 | 28(16-39) |

**Table D: Seroprevalence of DENV infection in general population.**

| **Regions** | **General population** | | | |
| --- | --- | --- | --- | --- |
|  | **Number of reports** | **cases** | **samples** | **seroprevalence(%,95%CI)** |
| **African** | 6 | 958 | 8766 | 13(7-20) |
| **Eastern Mediterranean** | 11 | 4490 | 15919 | 27(19-35) |
| **European** | 2 | 49 | 920 | 6(0-12) |
| **South-East Asia** | 5 | 3680 | 7504 | 56(14-97) |
| **the Americas** | 11 | 7599 | 11495 | 54(29-79) |
| **Western Pacific** | 11 | 5839 | 22992 | 26(14-37) |

**Table E: Seroprevalence of DENV infection stratified by diagnostic tests.**

| **Regions** | **IgG** | | | | **IgM** | | | |
| --- | --- | --- | --- | --- | --- | --- | --- | --- |
|  | **Number of reports** | **cases** | **samples** | **seroprevalence(%,95%CI)** | **Number of reports** | **cases** | **samples** | **seroprevalence(%,95%CI)** |
| **African** | 8 | 1066 | 10199 | 13(8-17) | 3 | 112 | 3235 | 4(-3-10) |
| **Eastern Mediterranean** | 14 | 5042 | 17121 | 29(23-34) | 2 | 91 | 1701 | 5(4-6) |
| **European** | 3 | 56 | 2100 | 4(0-7) | 1 | 1 | 562 | 0(-0-1) |
| **South-East Asia** | 15 | 15187 | 27381 | 59(47-70) | 3 | 54 | 1644 | 4(1-7) |
| **the Americas** | 27 | 14595 | 24807 | 54(40-69) | 10 | 382 | 4634 | 6(3-9) |
| **Western Pacific** | 20 | 8831 | 31429 | 28(19-36) | 8 | 585 | 16867 | 4(3-5) |

**Table F: Seroprevalence of DENV infection stratified by age.**

| **Regions** | **Children** | | | | **Adults** | | | |
| --- | --- | --- | --- | --- | --- | --- | --- | --- |
|  | **Number of reports** | **cases** | **Samples** | **seroprevalence(%,95%CI)** | **Number of reports** | **cases** | **Samples** | **seroprevalence(%,95%CI)** |
| **African** | 3 | 253 | 1945 | 13(0-28) | 1 | 162 | 1141 | 14(12-16) |
| **Eastern Mediterranean** | 1 | 100 | 400 | 25(21-29) | 3 | 1174 | 3603 | 37(22-52) |
| **European** | / | / | / | / | / | / | / | / |
| **South-East Asia** | 6 | 4493 | 7781 | 50(34-66) | 1 | 118 | 200 | 59(52-66) |
| **the Americas** | 8 | 1969 | 5087 | 42(22-63) | 5 | 1355 | 1954 | 56(8-100) |
| **Western Pacific** | 3 | 711 | 2698 | 30(5-56) | 8 | 5065 | 10710 | 40(19-61) |

**Table G: Proportion of DENV inapparent infection.**

| **Regions** | **Number of reports** | **cases** | **samples** | **inapparent proportion(%,95%CI)** |
| --- | --- | --- | --- | --- |
| **African** | 2 | 156 | 271 | 58(51-66) |
| **Eastern Mediterranean** | 1 | 324 | 1710 | 19(17-21) |
| **European** | / | / | / | / |
| **South-East Asia** | 7 | 4193 | 5719 | 93(89-98) |
| **the Americas** | 8 | 5363 | 6382 | 80(71-89) |
| **Western Pacific** | 6 | 767 | 849 | 90(83-96) |
| **Overall** | 24 | 10803 | 14931 | 80(72-88) |
