# Supplementary material for: The worldwide seroprevalence of DENV, CHIKV and ZIKV infection: A systematic review and meta-analysis
Source: PLoS Negl Trop Dis. 2021 Apr 28;15(4):e0009337. doi: 10.1371/journal.pntd.0009337 (PMC8109817; doi:10.1371/journal.pntd.0009337)
Supplement: S3 Text — Table A. Seroprevalence of CHIKV infection for developing and developed countries. Table B. Time trend of CHIKV seroprevalence. Table C. Seroprevalence of CHIKV infection in general population. Table D. Seroprevalence of CHIKV infection stratified by diagnostic tests. Table E. Seroprevalence of CHIKV infection for urban and rural areas. Table F. Proportion of CHIKV inapparent infection. (DOCX) [file pntd.0009337.s003.docx]

S3 Appendix

Table A: Seroprevalence of CHIKV infection for developing and developed countries.

Table B: Time trend of CHIKV seroprevalence.

Table C: Seroprevalence of CHIKV infection in general population.

Table D: Seroprevalence of CHIKV infection stratified by diagnostic tests .

Table E: Seroprevalence of CHIKV infection for urban and rural areas .

Table F: Proportion of CHIKV inapparent infection .

**Table A: Seroprevalence of CHIKV infection for developing and developed countries.**

| **Regions** | **Developing** | | | | **Developed** | | | |
| --- | --- | --- | --- | --- | --- | --- | --- | --- |
|  | **Number of reports** | **cases** | **samples** | **seroprevalence(%,95%CI)** | **Number of reports** | **cases** | **samples** | **seroprevalence(%,95%CI)** |
| **African** | 12 | 1894 | 8875 | 33(24-41) | / | / | / | / |
| **Eastern Mediterranean** | 3 | 108 | 3305 | 2(0-5) | / | / | / | / |
| **European** | 1 | 2 | 500 | 0(0-1) | 2 | 42 | 1333 | 5(0-14) |
| **South-East Asia** | 6 | 1291 | 4924 | 42(17-67) | / | / | / | / |
| **the Americas** | 8 | 3551 | 10877 | 25(10-40) | 3 | 418 | 1907 | 19(0-39) |
| **Western Pacific** | 3 | 200 | 4784 | 7(2-12) | / | / | / | / |

**Table B: Time trend of CHIKV seroprevalence.**

| **Regions** | **2000-2009** | | | | **2010-2019** | | | |
| --- | --- | --- | --- | --- | --- | --- | --- | --- |
|  | **Number of reports** | **cases** | **samples** | **seroprevalence(%,95%CI)** | **Number of reports** | **cases** | **samples** | **seroprevalence(%,95%CI)** |
| **African** | 10 | 3220 | 9876 | 39(27-51) | 4 | 2523 | 13085 | 19(6-32) |
| **Eastern Mediterranean** | / | / | / | / | 3 | 108 | 3305 | 2(0-5) |
| **European** | 1 | 33 | 325 | 10(7-13) | 2 | 11 | 1508 | 1(0-1) |
| **South-East Asia** | 3 | 401 | 2760 | 37(0-82) | 2 | 663 | 1845 | 35(19-51) |
| **the Americas** | / | / | / | / | 12 | 4011 | 12987 | 23(13-34) |
| **Western Pacific** | 1 | 56 | 945 | 6(4-7) | 2 | 144 | 3839 | 8(0-19) |

**Table C: Seroprevalence of CHIKV infection in general population**.

| **Regions** | **General population** | | | |
| --- | --- | --- | --- | --- |
|  | **Number of reports** | **cases** | **samples** | **seroprevalence(%,95%CI)** |
| **African** | 10 | 2963 | 9908 | 41(29-54) |
| **Eastern Mediterranean** | 3 | 108 | 3305 | 2(0-5) |
| **European** | 1 | 33 | 325 | 10(7-13) |
| **South-East Asia** | 2 | 324 | 2561 | 36(0-100) |
| **the Americas** | 8 | 3461 | 7703 | 28(8-49) |
| **Western Pacific** | 1 | 71 | 3293 | 2(2-3) |

**Table D: Seroprevalence of CHIKV infection stratified by diagnostic tests.**

| **Regions** | **IgG** | | | | **IgM** | | | |
| --- | --- | --- | --- | --- | --- | --- | --- | --- |
|  | **Number of reports** | **cases** | **samples** | **seroprevalence(%,95%CI)** | **Number of reports** | **cases** | **samples** | **seroprevalence(%,95%CI)** |
| **African** | 15 | 5494 | 23510 | 30(22-37) | 7 | 581 | 4362 | 24(15-33) |
| **Eastern Mediterranean** | 3 | 108 | 3305 | 2(0-5) | / | / | / | / |
| **European** | 3 | 44 | 1833 | 3(1-5) | / | / | / | / |
| **South-East Asia** | 5 | 1223 | 2744 | 49(33-66) | 2 | 98 | 518 | 16(0-38) |
| **the Americas** | 11 | 3930 | 12620 | 24(12-36) | 4 | 153 | 1521 | 8(0-15) |
| **Western Pacific** | 3 | 200 | 4784 | 7(2-12) | / | / | / | / |

**Table E: Seroprevalence of CHIKV infection for urban and rural areas.**

| **Regions** | **Rural** | | | | **Urban** | | | |
| --- | --- | --- | --- | --- | --- | --- | --- | --- |
|  | **Number of reports** | **cases** | **samples** | **seroprevalence(%,95%CI)** | **Number of reports** | **cases** | **samples** | **seroprevalence(%,95%CI)** |
| **African** | 6 | 1122 | 4784 | 42(22-63) | 3 | 287 | 1596 | 42(6-78) |
| **Eastern Mediterranean** | / | / | / | / | / | / | / | / |
| **European** | / | / | / | / | 1 | 33 | 325 | 10(7-13） |
| **South-East Asia** | 1 | 260 | 381 | 68(64-73) | / | / | / | / |
| **the Americas** | 2 | 24 | 120 | 33(8-57) | 3 | 429 | 1198 | 44(0-99） |
| **Western Pacific** | 1 | 40 | 386 | 10(7-13) | 2 | 71 | 3293 | 2(2-3） |

**Table F: Proportion of CHIKV inapparent infection.**

| **Regions** | **Number of reports** | **cases** | **samples** | **inapparent proportion(%,95%CI)** |
| --- | --- | --- | --- | --- |
| **African** | 5 | 455 | 1975 | 26(17-35) |
| **Eastern Mediterranean** | / | / | / | / |
| **European** | 1 | 6 | 33 | 18(5-31) |
| **South-East Asia** | 3 | 488 | 926 | 53(0-100) |
| **the Americas** | 7 | 574 | 1091 | 48(35-61) |
| **Western Pacific** | / | / | / | / |
| **Overall** | 16 | 1523 | 4025 | 40(24-56) |
