# Supplementary material for: The worldwide seroprevalence of DENV, CHIKV and ZIKV infection: A systematic review and meta-analysis
Source: PLoS Negl Trop Dis. 2021 Apr 28;15(4):e0009337. doi: 10.1371/journal.pntd.0009337 (PMC8109817; doi:10.1371/journal.pntd.0009337)
Supplement: S4 Text — Table A. The publications reporting cross-infection between any two of three arboviruses. Table B. The publications reporting both results of nucleic acid and antibody. Fig A. Forest plot of the pooled seroprevalence of cross-infection. (DOCX) [file pntd.0009337.s004.docx]

S4 Appendix

Table A: The publications reporting cross-infection between any two of three arboviruses.

Table B: The publications reporting both results of nucleic acid and antibody.

Fig A: Forest plot of the pooled seroprevalence of cross-infection.

**Table A: The publications reporting cross-infection between any two of three arboviruses .**

| **First author(Publication Year)** | **Cross-infection** | **cases** | **samples** | **seroprevalence** |
| --- | --- | --- | --- | --- |
| Gabor(2016) | DENV/CHIKV | 1 | 162 | 0.6 |
| Laoprasopwattana(2016) |  | 2 | 319 | 0.6 |
| Lozier(2018) |  | 3 | 367 | 0.8 |
| Vongpunsawad(2017) |  | 204 | 835 | 24.4 |
| Schwarz(2012) |  | 22 | 1244 | 1.8 |
| Rodriguez-Barraquer(2015) |  | 325 | 800 | 41.0 |
| Lozier(2018) | CHIKV/ZIKA | 1 | 367 | 0.3 |
| Willcox(2018) | DENV/ZIKA | 17 | 978 | 1.7 |

**Table B: The publications reporting both results of nucleic acid and antibody.**

| **First author(Publication Year)** | **arbovirus** | **Nucleic acid** | | | **Antibody** | | |
| --- | --- | --- | --- | --- | --- | --- | --- |
|  |  | **cases** | **samples** | **seroprevalence** | **cases** | **samples** | **seroprevalence** |
| Alayed(2018) | ZIKA | 0 | 52 | 0 | 52 | 410 | 12.7 |
| Ellis(2015) |  | 101 | 1500 | 6.7 | 210 | 1500 | 14.0 |
| Lozier(2018) |  | 27 | 367 | 7.4 | 79 | 367 | 21.5 |
| Lozier(2018) | CHIKV | 0 | 367 | 0 | 5 | 367 | 1.4 |
| Slavov(2018) |  | 0 | 442 | 0 | 1 | 442 | 0.2 |
| Lozier(2018) | DENV | 0 | 367 | 0 | 3 | 367 | 0.8 |
| Ranjan(2016) |  | 0 | 200 | 0 | 118 | 200 | 59.0 |
| Slavov(2019) |  | 0 | 475 | 0 | 32 | 475 | 6.7 |

**Fig A: Forest plot of the pooled seroprevalence of cross-infection.**
