# Supplementary material for: The worldwide seroprevalence of DENV, CHIKV and ZIKV infection: A systematic review and meta-analysis
Source: PLoS Negl Trop Dis. 2021 Apr 28;15(4):e0009337. doi: 10.1371/journal.pntd.0009337 (PMC8109817; doi:10.1371/journal.pntd.0009337)
Supplement: S5 Text — Table A. Assessments of the quality of the studies. (DOC) [file pntd.0009337.s005.doc]

S5 Appendix

Table A: Assessments of the quality of the studies.

Table A: Assessments of the quality of the studies.

| **ID** | **1) Define the source of information (survey, record review)** | **2) List inclusion and exclusion criteria for exposed and unexposed subjects (cases and controls) or refer to previous publications** | **3) Indicate time period used for identifying patients** | **4) Indicate whether or not subjects were consecutive if not population-based** | **5) Indicate if evaluators of subjective components of study were masked to other aspects of the status of the participants** | **6) Describe any assessments undertaken for quality assurance purposes (e.g., test/retest of primary outcome measurements)** | **7) Explain any patient exclusions from analysis** | **8) Describe how confounding was assessed and/or controlled.** | **9) If applicable, explain how missing data were handled in the analysis** | **10) Summarize patient response rates and completeness of data collection** | **11) Clarify what follow-up, if any, was expected and the percentage of patients for which incomplete data or follow-up was obtained** | **Total Score** | **Bias quality** |
| --- | --- | --- | --- | --- | --- | --- | --- | --- | --- | --- | --- | --- | --- |
| 1 | Yes | Unclear | Yes | No | No | Yes | No | Yes | No | Yes | No | 5 | M |
| 2 | Yes | No | No | No | No | Yes | No | Yes | Yes | No | No | 4 | M |
| 3 | Yes | Yes | Yes | Yes | No | Yes | No | No | No | Yes | No | 6 | M |
| 4 | Yes | Yes | Yes | Yes | No | Yes | Yes | Unclear | No | Yes | No | 7 | M |
| 5 | Yes | No | Yes | Unclear | Yes | Yes | No | Yes | No | Yes | No | 6 | M |
| 6 | Yes | No | Yes | Unclear | Yes | Yes | No | Yes | No | Yes | No | 6 | M |
| 7 | Yes | Yes | Yes | No | No | Yes | No | No | No | Yes | No | 5 | M |
| 8 | Yes | Yes | Yes | No | No | Yes | No | Unclear | Yes | Yes | No | 6 | M |
| 9 | Yes | No | Yes | No | No | Yes | No | No | Yes | Yes | No | 5 | M |
| 10 | Yes | Yes | Yes | Unclear | No | Yes | No | Yes | Yes | No | No | 6 | M |
| 11 | Yes | No | Yes | Yes | No | Yes | No | Yes | No | No | No | 5 | M |
| 12 | Yes | No | Yes | No | No | Yes | No | Unclear | Yes | No | No | 4 | M |
| 13 | Yes | Yes | Yes | Yes | No | Yes | No | No | No | Yes | No | 6 | M |
| 14 | Yes | Yes | Yes | Yes | No | Yes | Yes | Yes | Yes | Yes | No | 9 | H |
| 15 | Yes | No | Yes | No | No | Yes | No | No | Yes | No | No | 4 | M |
| 16 | Yes | Yes | Yes | Yes | No | Yes | No | Yes | Yes | Yes | Yes | 9 | H |
| 17 | Yes | Yes | Yes | Yes | No | Yes | Yes | Yes | Yes | Yes | No | 9 | H |
| 18 | Yes | Yes | Yes | No | No | Yes | No | Yes | No | Yes | No | 6 | M |
| 19 | Yes | No | Yes | No | No | Yes | No | No | Yes | No | No | 4 | M |
| 20 | Yes | Yes | Yes | Yes | No | Yes | No | No | No | No | No | 5 | M |
| 21 | Yes | No | Yes | Yes | No | Yes | Yes | No | No | Yes | No | 6 | M |
| 22 | Yes | No | Yes | No | No | Yes | No | No | Yes | No | No | 4 | M |
| 23 | Yes | Yes | Yes | No | No | Yes | Yes | No | No | Yes | No | 6 | M |
| 24 | Yes | Yes | Yes | No | No | Yes | No | No | Yes | Yes | No | 6 | M |
| 25 | Yes | Yes | Yes | No | No | Yes | Unclear | Yes | No | No | No | 5 | M |
| 26 | Yes | Yes | Yes | No | No | Yes | No | Yes | Yes | Yes | No | 7 | M |
| 27 | Yes | Yes | Yes | Yes | No | Yes | Yes | No | Yes | Yes | No | 8 | H |
| 28 | Yes | Yes | Yes | No | No | Yes | No | Yes | Yes | Yes | No | 7 | M |
| 29 | Yes | Yes | Yes | No | No | Yes | No | Yes | Yes | Yes | No | 7 | M |
| 30 | Yes | No | Yes | No | No | Yes | No | Yes | Yes | No | No | 5 | M |
| 31 | Yes | No | Yes | Yes | No | Yes | No | No | No | Yes | No | 5 | M |
| 32 | Yes | No | Yes | No | Unclear | No | No | No | Yes | No | No | 3 | L |
| 33 | Yes | Yes | Yes | Yes | No | Yes | Yes | Yes | Yes | Yes | No | 8 | H |
| 34 | Yes | No | Yes | Yes | No | Yes | Yes | No | Yes | Yes | No | 6 | M |
| 35 | Yes | No | Yes | Yes | Yes | Unclear | No | Unclear | No | Yes | No | 5 | M |
| 36 | Yes | No | Yes | Yes | No | Yes | No | Unclear | Yes | No | No | 5 | M |
| 37 | Yes | Yes | Yes | Yes | No | Yes | Yes | Yes | No | Yes | No | 8 | H |
| 38 | Yes | No | Yes | Yes | No | Yes | No | Yes | No | No | No | 4 | M |
| 39 | Yes | Yes | Yes | Yes | Yes | Yes | No | No | No | Yes | No | 5 | M |
| 40 | Yes | Yes | Yes | Yes | Unclear | Yes | Yes | No | Yes | Yes | No | 8 | H |
| 41 | Yes | Yes | No | Yes | Yes | Yes | No | No | No | No | No | 4 | M |
| 42 | Yes | Yes | Yes | Yes | No | Yes | Yes | Yes | Yes | Yes | No | 9 | H |
| 43 | Yes | Yes | Yes | Yes | No | Yes | Yes | Yes | Yes | Yes | No | 9 | H |
| 44 | Yes | Unclear | Yes | Yes | No | Yes | Yes | Yes | Yes | Yes | No | 8 | H |
| 45 | Yes | No | Yes | Yes | No | Yes | Yes | Unclear | Yes | Yes | No | 7 | M |
| 46 | Yes | No | Yes | Yes | No | Yes | No | Yes | No | No | No | 6 | M |
| 47 | Yes | Unclear | Yes | Yes | No | Yes | No | No | Yes | No | No | 5 | M |
| 48 | Yes | Yes | Yes | Yes | No | Yes | Yes | No | Yes | Yes | No | 8 | H |
| 49 | Yes | Unclear | Unclear | Yes | No | Yes | No | No | No | Yes | No | 4 | M |
| 50 | Yes | Yes | Yes | Yes | Yes | Yes | No | No | No | No | No | 5 | M |
| 51 | Yes | Yes | Yes | Yes | No | Yes | Yes | Yes | Yes | Yes | No | 9 | H |
| 52 | Yes | Unclear | Yes | Yes | No | Yes | Yes | Yes | Yes | Yes | No | 8 | H |
| 53 | Yes | Yes | Yes | Yes | No | Yes | Yes | No | Yes | Yes | No | 8 | H |
| 54 | Yes | No | Yes | Yes | No | Yes | No | Yes | No | No | No | 5 | M |
| 55 | Yes | Yes | Yes | Yes | Yes | Yes | Yes | Yes | Yes | Yes | No | 10 | H |
| 56 | Yes | Yes | Yes | Yes | No | Yes | Yes | Yes | No | Yes | No | 7 | M |
| 57 | Yes | Unclear | Yes | Yes | No | Yes | No | Yes | Yes | Yes | No | 7 | M |
| 58 | Yes | Unclear | Unclear | Yes | No | Yes | No | No | No | No | No | 3 | L |
| 59 | Yes | Yes | No | Yes | No | Yes | Yes | No | No | Yes | Yes | 8 | H |
| 60 | Yes | Yes | Yes | Yes | No | Yes | No | No | Yes | No | No | 6 | M |
| 61 | Yes | Yes | No | Yes | No | Yes | Yes | Yes | Yes | No | No | 7 | M |
| 62 | Yes | Yes | Yes | Yes | Unclear | Yes | No | Yes | No | Yes | Unclear | 7 | M |
| 63 | Yes | Yes | Yes | Yes | Unclear | Yes | Yes | Yes | Yes | Yes | Unclear | 9 | H |
| 64 | Yes | Yes | Yes | Yes | Unclear | Yes | No | No | No | No | Unclear | 5 | M |
| 65 | Yes | No | Yes | Yes | Yes | Yes | Yes | No | Yes | Yes | Unclear | 8 | H |
| 66 | Yes | Yes | No | Yes | Unclear | Yes | Yes | No | Yes | Yes | Unclear | 7 | M |
| 67 | Yes | No | Yes | Yes | Unclear | Yes | Yes | Yes | Yes | Yes | Unclear | 8 | H |
| 68 | Yes | Yes | Yes | Yes | No | Yes | Yes | No | Yes | Yes | Unclear | 8 | H |
| 69 | Yes | Yes | No | No | No | No | No | No | Unclear | No | Unclear | 2 | L |
| 70 | Yes | No | Yes | Unclear | No | Yes | No | Yes | No | No | Unclear | 4 | M |
| 71 | Yes | No | Yes | Yes | No | Yes | No | Yes | No | No | No | 5 | M |
| 72 | Yes | Yes | Yes | Yes | No | Yes | Yes | Yes | Yes | Yes | No | 9 | H |
| 73 | Yes | Yes | Yes | Unclear | Unclear | Yes | Unclear | No | Unclear | No | No | 4 | M |
| 74 | Yes | Yes | Yes | Yes | No | Yes | No | Yes | No | No | No | 6 | M |
| 75 | Yes | No | Yes | Yes | Yes | Yes | No | No | No | No | No | 5 | M |
| 76 | Yes | Yes | Yes | Yes | No | Yes | Yes | Yes | Yes | Yes | Yes | 10 | H |
| 77 | Yes | Yes | Yes | Yes | No | Yes | Yes | Yes | Yes | Yes | Unclear | 9 | H |
| 78 | Yes | Yes | Yes | Unclear | Unclear | Yes | Yes | No | Yes | Yes | No | 7 | M |
| 79 | Yes | Yes | Yes | Unclear | Unclear | Yes | Yes | No | Yes | Yes | No | 7 | M |
| 80 | Yes | Yes | Yes | Unclear | No | Yes | Yes | Yes | Yes | Yes | Yes | 9 | H |
| 81 | Yes | Yes | Yes | Unclear | No | Yes | Yes | No | Yes | Yes | No | 7 | M |
| 82 | Yes | Yes | Yes | Yes | No | Yes | Yes | Yes | Yes | Yes | No | 9 | H |
| 83 | Yes | No | No | Unclear | Yes | Yes | Unclear | Unclear | Unclear | No | No | 3 | L |
| 84 | Yes | No | Yes | Yes | Unclear | Yes | Yes | No | Yes | Yes | Unclear | 6 | M |
| 85 | Yes | Yes | Yes | Yes | No | Yes | Yes | Yes | Yes | Yes | No | 9 | H |
| 86 | Yes | No | Yes | Unclear | No | Yes | Yes | No | Yes | Yes | No | 6 | M |
| 87 | Yes | Yes | Yes | Unclear | No | Yes | Yes | Yes | Yes | Yes | Yes | 9 | H |
| 88 | Yes | No | Yes | Unclear | Unclear | Yes | Yes | No | Yes | Yes | No | 6 | M |
| 89 | Yes | No | Yes | Yes | No | Yes | Yes | Yes | Yes | Yes | No | 8 | H |
| 90 | Yes | Yes | Yes | Yes | No | Yes | No | Yes | No | No | No | 6 | M |
| 91 | Yes | No | Yes | Unclear | No | Yes | Yes | Yes | Yes | Yes | No | 7 | M |
| 92 | Yes | Yes | Yes | Yes | No | Yes | Yes | Yes | Yes | Yes | No | 9 | H |
| 93 | Yes | No | Yes | Yes | No | Yes | Yes | Yes | No | No | No | 6 | M |
| 94 | Yes | No | Yes | Yes | No | Yes | Yes | No | Yes | No | No | 6 | M |
| 95 | Yes | Yes | Yes | Unclear | No | Yes | No | Yes | No | No | No | 5 | M |
| 96 | Yes | Yes | Yes | Yes | No | Yes | Yes | Unclear | Yes | Yes | No | 8 | H |
| 97 | Yes | Yes | Yes | Yes | No | Yes | Yes | Unclear | Yes | Yes | No | 8 | H |
| 98 | Yes | Yes | Yes | Yes | Yes | Yes | Yes | No | Yes | Yes | No | 9 | H |
| 99 | Yes | Yes | Yes | Yes | No | Yes | Yes | No | Yes | Yes | Yes | 9 | H |
| 100 | Yes | Yes | Yes | Yes | Unclear | Yes | Yes | No | Yes | Yes | No | 8 | H |
| 101 | Yes | Yes | Yes | Yes | No | Yes | Yes | No | Yes | Yes | No | 8 | H |
| 102 | Yes | Yes | Yes | Yes | No | Yes | Yes | Yes | Yes | Yes | No | 9 | H |
| 103 | Yes | Yes | Yes | Yes | No | Yes | Yes | Yes | Yes | Yes | No | 9 | H |
| 104 | Yes | Yes | Yes | Unclear | Unclear | Yes | Yes | Unclear | Unclear | Yes | No | 6 | M |
| 105 | Yes | No | Yes | Unclear | Unclear | Yes | Unclear | No | Yes | Unclear | No | 3 | L |
| 106 | Yes | Yes | Yes | Yes | No | Yes | Yes | Yes | Yes | Yes | No | 9 | H |
| 107 | Yes | Yes | Yes | Yes | No | Yes | Yes | Yes | Yes | Yes | No | 9 | H |
| 108 | Yes | Yes | Yes | No | No | Yes | Yes | No | Yes | Yes | No | 7 | M |
| 109 | Yes | Yes | Yes | Yes | Unclear | Yes | Yes | No | Yes | Yes | Yes | 9 | H |
| 110 | Yes | Yes | No | Unclear | Unclear | Yes | Yes | No | Yes | Yes | No | 6 | M |
| 111 | Yes | No | Yes | No | No | Yes | No | Unclear | Yes | No | No | 4 | M |
| 112 | Yes | Yes | Yes | Yes | Unclear | Yes | No | No | No | No | Unclear | 5 | M |
| 113 | Yes | No | Yes | Yes | No | Yes | Yes | Yes | Yes | Yes | Yes | 9 | H |
| 114 | Yes | Yes | Yes | Unclear | No | Yes | Yes | Yes | Yes | Yes | No | 8 | H |
| 115 | Yes | No | Yes | Yes | Unclear | Yes | Unclear | No | Unclear | Unclear | No | 4 | M |
| 116 | Yes | Yes | Yes | Unclear | Yes | Yes | Yes | No | Yes | Yes | No | 8 | H |
| 117 | Yes | No | Yes | Yes | No | Yes | Yes | Yes | Yes | Yes | No | 8 | H |
| 118 | Yes | Yes | Yes | Unclear | No | Yes | Yes | Yes | Yes | Yes | No | 8 | H |
| 119 | Yes | Yes | No | Unclear | No | Yes | Yes | No | Yes | Yes | No | 6 | M |
| 120 | Yes | No | Yes | Yes | Unclear | Yes | Yes | No | Yes | Yes | No | 7 | M |
| 121 | Yes | Yes | Yes | Unclear | No | Yes | Yes | No | Yes | Yes | No | 7 | M |
| 122 | Yes | Yes | Yes | Yes | No | Yes | Yes | Yes | Yes | Yes | No | 9 | H |
| 123 | Yes | Yes | Yes | No | No | Yes | No | Yes | Yes | Yes | No | 7 | M |
| 124 | Yes | Yes | Yes | Unclear | No | Yes | No | Yes | Yes | No | No | 6 | M |
| 125 | Yes | No | Yes | No | Unclear | Yes | Yes | No | Unclear | Unclear | No | 4 | M |
| 126 | Yes | No | No | Yes | No | Yes | Yes | Yes | No | No | No | 5 | M |
| 127 | Yes | No | Yes | No | No | Yes | Yes | Yes | No | Yes | No | 6 | M |
| 128 | Yes | Yes | Yes | No | No | Yes | Yes | Yes | Yes | Yes | No | 8 | H |
| 129 | Yes | Yes | Yes | Yes | No | Yes | Yes | Yes | Yes | Yes | No | 9 | H |
| 130 | Yes | Yes | Yes | Yes | No | Yes | Yes | Yes | Yes | Yes | No | 9 | H |
| 131 | Yes | Yes | Yes | Yes | No | Yes | Yes | Unclear | Yes | Yes | Yes | 9 | H |
| 132 | Yes | No | Yes | Unclear | Yes | Yes | No | No | Yes | No | No | 5 | M |
| 133 | Yes | No | No | Yes | Unclear | Yes | Yes | No | Yes | Yes | No | 6 | M |
